# Correction: A randomised controlled feasibility trial of a BabyWASH household playspace: The CAMPI study

**DOI:** 10.1371/journal.pntd.0013351

**Published:** 2025-07-24

**Authors:** Sophie Budge, Paul Hutchings, Alison Parker, Sean Tyrrel, Sam Norton, Camila Garbutt, Fitsume Woldemedhin, Mohammed Yasin Jemal, Mathewos Moges, Siraj Hussen, Hunachew Beyene

The second author, Paul Hutchings, is incorrectly noted as the corresponding author. The correct corresponding author is Alison Parker. Alison Parker’s email address is: a.parker@cranfield.ac.uk.
