# Supplementary material for: A randomised controlled feasibility trial of a BabyWASH household playspace: The CAMPI study
Source: PLoS Negl Trop Dis. 2021 Jul 14;15(7):e0009514. doi: 10.1371/journal.pntd.0009514 (PMC8312948; doi:10.1371/journal.pntd.0009514)
Supplement: S1 Text — A description of the modified barrier analysis methodology including questions used to assess behavioural barriers to appropriate use and maintenance of the BabyWASH household playspace. (DOCX) [file pntd.0009514.s002.docx]

**S2.** **Modified Barrier Analysis Methodology**

**Background to a Barrier Analysis**

A Barrier Analysis (BA) is the main type of formative research recommended in the Designing for Behavioural Change framework. It is described as the ‘field research step’ with a primary aim to identify what is preventing the target groups from practicing the targeted behaviours (the ‘barriers’) and what might encourage adoption (the ‘motivators’).^1^ The full official training syllabus, published by Kittle BL, provides a practical guide.^2^ Briefly, the standard BA method asks the target group a series of questions aiming to identify barriers and motivators, using a Doer/Non-Doer methodology. This consists of interviewing the group who already do the behaviour and the group who have not yet adopted the behaviour. The motivators and barriers identified in the survey are categorized under 1 of 12 possible determinants. These determinants represent the perceptions, feelings, and beliefs of the target group and determine why they do or do not adopt a specific behaviour and are as follows: Perceived self-efficacy; Perceived social norms; Perceived positive consequences; Perceived negative consequences; Access; Cues for action/reminders; Perceived susceptibility/risk; Perceived severity; Perceived action efficacy; Perceived divine will; Policy and Culture. Analysing the difference between the Doers’ and Non-Doers’ responses indicates which barriers or enabling factors are the most important in determining that behaviour. Insights into these perceptions are used to further develop the content and strategies of the behavioural change activity. Such links are called ‘Bridges to Activities’.

**Barrier Analysis method in the CAMPI feasibility trial**

Given that there were no ‘non-doers’ in the CAMPI feasibility trial, intervention households were not analysed according to the standard Doer/Non-doer methodology as described above. Rather, the methodology was modified slightly to explore behaviours among all participants, without categorising them as Doers/Non-doers. The survey used in the modified BA is shown below. Intervention households were interviewed by the data collection teams, trained in the BA method, alongside a Health Extension Worker at week four of the feasibility trial. Following data collection, responses were entered into Excel. Questions 2−9 were entered into categories according to determinants and then categorised into themes which arose from the data. Those themes were summed and described in the results. Quantitative responses (questions 1, 10−16 in the table below) were summed in each answer category and also subsequently described in the result. As such, the determinants allowed for the description of barriers/enabling factors among all households which would improve adherence to a greater degree – given that all households used the playspace during the trial.

| 1 | Do you think that you use the play space for your child whenever you can? | Yes |
| --- | --- | --- |
|  |  | No |
| 2 | What are the **advantages** of using the play space? |  |
| 3 | What are the **disadvantages** of using the play space? |  |
| 4 | What makes it **easy** for you to use the play space? |  |
| 5 | What makes it **difficult** for you to use the play space? |  |
| 6 | What makes it **easy** for you to keep the play space clean? |  |
| 7 | What makes it **difficult** for you to keep the play space clean? |  |
| 8 | Who are the people who **approve** of you using the play space for your child? |  |
| 9 | Who are the people who **disapprove** of you using the play space for your child? |  |
| 10 | How difficult is it to remember to use the play space for your child every time you could use it? | Very difficult |
|  |  | A bit difficult |
|  |  | Not difficult |
|  |  | Don’t know |
| 11 | How likely do you think it is your child will get diarrhoeal disease within the next month? | Very likely |
|  |  | Quite likely |
|  |  | Not likely |
|  |  | Don’t know |
| 12 | How serious would it be if your child had a diarrhoeal disease? | Very serious |
|  |  | Quite serious |
|  |  | Not serious |
|  |  | Don’t know |
| 13 | How likely do you think it is your child will get diarrhoeal disease if you used the play space whenever you could? | Very likely |
|  |  | Quite likely |
|  |  | Not likely |
|  |  | Don’t know |
| 14 | Do you think God approves of you using the play space? | Yes |
|  |  | No |
|  |  | Don’t know |
|  |  | Specify: |
| 15 | Are there any community rules which prevent you from using the play space?  *If yes,* what are they? | Yes |
|  |  | No |
|  |  | Don’t know |
|  |  | Specify: |
| 16 | Are there any cultural rules that you know of against using the play space?  *If yes,* what are they? | Yes |
|  |  | No |
|  |  | Don’t know |
|  |  | Specify: |
| 17 | Ask the caregiver:  *Do you have any other comments about the play space, positive* ***OR*** *negative?* |  |

1. Schmied, P 2017. Behavioural Change Toolkit for International Development Practitioners. People In Need: Prague, Czech Republic.

2. Kittle, B. 2017. A Practical Guide to Conducting a Barrier Analysis (2nd ed.). Helen Keller International: New York, NY.
