# Supplementary figures and images for: A randomised controlled feasibility trial of a BabyWASH household playspace: The CAMPI study
Source: PLoS Negl Trop Dis. 2021 Jul 14;15(7):e0009514. doi: 10.1371/journal.pntd.0009514 (PMC8312948; doi:10.1371/journal.pntd.0009514)

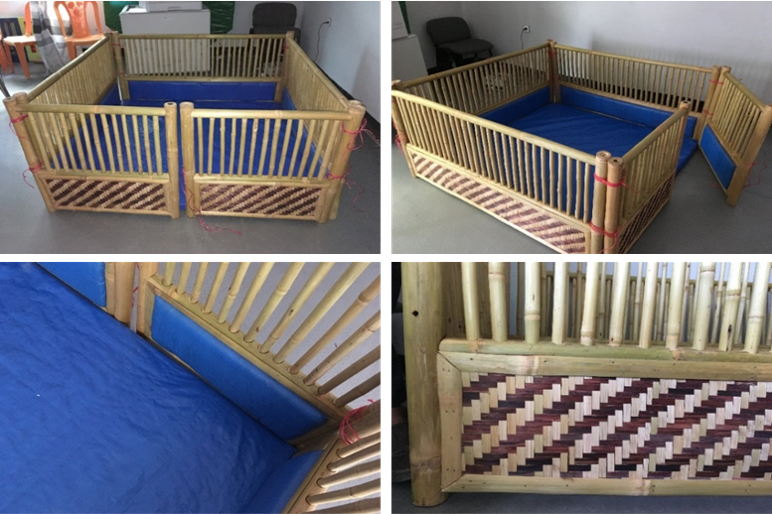

Supplement: S1 Fig — A figure containing four photographs showing the key design features of the BabyWASH household playspace. (TIF) [file pntd.0009514.s003.tif]
