# Supplementary material for: A randomised controlled feasibility trial of a BabyWASH household playspace: The CAMPI study
Source: PLoS Negl Trop Dis. 2021 Jul 14;15(7):e0009514. doi: 10.1371/journal.pntd.0009514 (PMC8312948; doi:10.1371/journal.pntd.0009514)
Supplement: S1 Data — A file containing supplementary data tables including: Table A. Playspace use behaviours and infant hygiene and playspace cleaning practices included as part of composite variables ‘Appropriate use’ and ‘Appropriate cleaning’. A table containing the full set of behavioural results on appropriate use and appropriate cleaning at two week, four week and both time points. Table B. Reported daily activities and reported use or non-use of the playspace during the past 24 hours, across daily periods and study time points. A table containing the full set of results on daily activities that BabyWASH playspace users engaged with whilst using the playspace at two week and four week time points. Table C. Reported playspace use and non-use during daily activities in the past 24 hours across daily time periods: at two and four weeks. A table containing results for reported use and non-use of the BabyWASH playspace during morning, afternoon and evenings. Table D. Modified Barrier Analysis results among the study intervention group. Two tables containing results from the modified barrier analysis covering key behavioural determinants, including perceived positive consequences, perceived self-efficacy, access and perceived social norms. Table E. Number of samples positive for presumptive Campylobacter spp. under each category of colony count. A table containing results for presumptive Campylobacter spp. colony count within intervention and control arm at baseline, two weeks and four weeks. Table F. Feeding of fresh or reheated foods prepared as recommended, across study groups and time points. A table containing results for reported feeding of fresh and reheated food at different meal times within intervention and control arm at baseline, two weeks and four weeks. Table G. Number of meals safely prepared across time points. A table containing results on number of safely prepared meals across intervention and control arm at baseline, two weeks and four weeks. (DOCX) [file pntd.0009514.s004.docx]

**S4 Supplementary Data tables**

**Table A. Playspace use behaviours and infant hygiene and playspace cleaning practices included as part of composite variables ‘Appropriate use’ and ‘Appropriate cleaning’.**

| **HPS use behaviours** | | | | | | |
| --- | --- | --- | --- | --- | --- | --- |
|  | Two weeks | | Four weeks | | Both time points | |
|  | n  (50) | % | n  (50) | % | N  (100)^*^ | %^**^ |
| Who watches the infant: Another child | 42 | 84.0 | 43 | 86.0 | 85 | 85.0 |
| Mother | 27 | 54.0 | 28 | 46.0 | 55 | 55.0 |
| Husband | 18 | 36.0 | 24 | 48.0 | 42 | 42.0 |
| A grandparent | 0 | 0.0 | 1 | 2.0 | 1 | 1.0 |
| Infant in HPS when leave house | 41 | 82.0 | 26 | 52.0 | 67 | 67.0 |
| Other child shares the HPS | 14 | 28.0 | 18 | 36.0 | 32 | 32.0 |
| Who shares the HPS: Mother to feed | 6 | 12.0 | 10 | 20.0 | 16 | 16.0 |
| Sister or brother | 4 | 8.0 | 4 | 8.0 | 8 | 8.0 |
| Another child | 2 | 4.0 | 6 | 12.0 | 8 | 8.0 |
| Twin | 1 | 2.0 | 1 | 2.0 | 2 | 2.0 |
| Infant given toys or items to play | 43 | 86.0 | 46 | 92.0 | 89 | 89.0 |
| Items given: Plastic cup | 32 | 64.0 | 33 | 66.0 | 65 | 65.0 |
| Plastic water bottle | 27 | 54.0 | 27 | 54.0 | 54 | 54.0 |
| Jerry can cover | 8 | 14.0 | 6 | 12.0 | 14 | 14.0 |
| Empty plastic container | 7 | 13.0 | 5 | 10.0 | 12 | 12.0 |
| Mobile phone | 6 | 12.0 | 6 | 12.0 | 12 | 12.0 |
| Small ball | 5 | 10.0 | 5 | 10.0 | 10 | 10.0 |
| Store-bought plastic toys | 2 | 4.0 | 6 | 12.0 | 8 | 8.0 |
| Book/paper | 2 | 4.0 | 2 | 4.0 | 4 | 4.0 |
| Reasons to remove infant: Infant hungry | 49 | 98.0 | 49 | 98.0 | 98 | 98.0 |
| Infant is crying (bored) | 44 | 88.0 | 46 | 92.0 | 90 | 90.0 |
| Infant has defecated/urinated | 39 | 78.0 | 37 | 74.0 | 76 | 76.0 |
| To clean the playspace | 30 | 60.0 | 26 | 52.0 | 56 | 56.0 |
| To wash/change infant | 25 | 50.0 | 30 | 60.0 | 55 | 55.0 |
| To breastfeed/feed | 11 | 22.0 | 15 | 30.0 | 26 | 26.0 |
| Infant is sleeping | 4 | 8.0 | 1 | 2.0 | 5 | 5.0 |
| To go out | 1 | 2.0 | 2 | 4.0 | 3 | 3.0 |
| **Infant hygiene and HPS cleaning** | | | | | | |
| **Observational data** | | | | | | |
| Infant visibly dirty upon arrival | 20 | 40.0 | 19 | 38.0 | 39 | 39.0 |
| Infant has dirty hands and nails | 28 | 56.0 | 24 | 48.0 | 52 | 52.0 |
| Visible dirt on mattress | 6 | 12.0 | 3 | 6.0 | 9 | 9.0 |
| Urine or faeces on mattress | 1 | 2.0 | 1 | 2.0 | 2 | 2.0 |
| Animals inside HPS (observed) | 1 | 2.0 | 0 | 0.0 | 1 | 1.0 |
| **Caregiver-reported data** | | | | | | |
| How often clean HPS: Every day | 30 | 60.0 | 16 | 32.0 | 46 | 46.0 |
| Twice a week | 9 | 18.0 | 17 | 34.0 | 26 | 26.0 |
| Every other day | 6 | 12.0 | 6 | 12.0 | 12 | 12.0 |
| Only when infant defecates/urinates | 3 | 6.0 | 11 | 22.0 | 14 | 14.0 |
| Only when it is dirty | 2 | 4.0 | 0 | 0.0 | 2 | 2.0 |
| Cleaning materials used: Water only | 5 | 10.0 | 2 | 4.0 | 7 | 7.0 |
| Water and soap | 45 | 90.0 | 48 | 96.0 | 93 | 93.0 |
| Animals seen inside HPS: Yes | 4 | 8.0 | 0 | 100.0 | 4 | 4.0 |
| Which animals? Cat | 2 | 50.0* | 0 | 0.0 | 2 | 2.0 |
| Poultry | 2 | 50.0* | 0 | 0.0 | 2 | 2.0 |
| HPS, household playspace.  ^*^Calculated as a cumulative total of both time points.  ^**^Percent is of the cumulative total. | | | | | | |

**Table B. Reported daily activities and reported use or non-use of the playspace during the past 24 hours, across daily periods and study time points.**

|  | **Morning** | | | | | | | | **Afternoon** | | | | | | | | **Evening** | | | | | | | | |
| --- | --- | --- | --- | --- | --- | --- | --- | --- | --- | --- | --- | --- | --- | --- | --- | --- | --- | --- | --- | --- | --- | --- | --- | --- | --- |
|  | **Two weeks** | | | | **Four weeks** | | | | **Two weeks** | | | | **Four weeks** | | | | **Two weeks** | | | | **Four weeks** | | | | |
|  | Used HPS | | Did not use HPS | | Used HPS | | Did not use HPS | | Used HPS | | Did not use HPS | | Used HPS | | Did not use HPS | | Used HPS | | Did not use HPS | | Used HPS | | Did not use HPS | | |
|  | n^*^ | %^**^ | n | % | n | % | n | % | n | % | n | % | n | % | n | % | n | % | n | % | n | % | n | % |  |
| Prepared breakfast | 40 | 89.0 | 5 | 11.0 | 42 | 89.0 | 5 | 11.0 | 0 | 0.0 | 0 | 0.0 | 0 | 0.0 | 0 | 0.0 | 0 | 0.0 | 0 | 0.0 | 0 | 0.0 | 0 | 0.0 |  |
| Prepared coffee | 32 | 94.0 | 2 | 6.0 | 37 | 95.0 | 2 | 5.0 | 5 | 83.0 | 1 | 17.0 | 14 | 88.0 | 2 | 12.0 | 32 | 91.0 | 3 | 9.0 | 34 | 85.0 | 6 | 15.0 |  |
| Cleaned the house | 37 | 86.0 | 6 | 14.0 | 35 | 83.0 | 7 | 17.0 | 3 | 75.0 | 1 | 25.0 | 9 | 100.0 | 0 | 0.0 | 0 | 0.0 | 0 | 0.0 | 3 | 75.0 | 1 | 25.0 |  |
| Fetched water | 22 | 96.0 | 1 | 4.0 | 17 | 85.0 | 3 | 15.0 | 18 | 100.0 | 0 | 0.0 | 26 | 96.0 | 1 | 4.0 | 0 | 0.0 | 0 | 0.0 | 1 | 100.0 | 0 | 0.0 |  |
| Prepared lunch/snacks | 0 | 0.0 | 0 | 0.0 | 0 | 0.0 | 0 | 0.0 | 48 | 96.0 | 2 | 4.0 | 44 | 98.0 | 1 | 2.0 | 6 | 100.0 | 0 | 0.0 | 4 | 67.0 | 2 | 33.0 |  |
| Prepared enset | 9 | 82.0 | 2 | 18.0 | 7 | 100.0 | 0 | 0.0 | 16 | 84.0 | 3 | 16.0 | 12 | 92.0 | 1 | 7.0 | 0 | 0.0 | 0 | 0.0 | 0 | 0.0 | 0 | 0.0 |  |
| Washed clothes | 2 | 50.0 | 2 | 50.0 | 1 | 33.0 | 2 | 67.0 | 4 | 67.0 | 2 | 33.0 | 12 | 82.0 | 1 | 7.0 | 0 | 0.0 | 0 | 0.0 | 2 | 100.0 | 0 | 0.0 |  |
| Farmed / maintained shop | 2 | 67.0 | 1 | 33.0 | 6 | 86.0 | 1 | 14.0 | 0 | 0.0 | 1 | 100.0 | 9 | 90.0 | 1 | 10.0 | 2 | 100.0 | 0 | 0.0 | 3 | 75.0 | 1 | 25.0 |  |
| Prepared dinner | 0 | 0.0 | 0 | 0.0 | 0 | 0.0 | 0 | 0.0 | 0 | 0.0 | 0 | 0.0 | 0 | 0.0 | 0 | 0.0 | 45 | 92.0 | 4 | 8.0 | 43 | 88.0 | 6 | 12.0 |  |
| Went to church / meeting | 1 | 100.0 | 0 | 0.0 | 0 | 0.0 | 4 | 100.0 | 0 | 0.0 | 3 | 100.0 | 0 | 0.0 | 0 | 0.0 | 0 | 0.0 | 0 | 0.0 | 0 | 0.0 | 0 | 0.0 |  |
| Went to market | 1 | 100.0 | 0 | 0.0 | 0 | 0.0 | 0 | 0.0 | 13 | 93.0 | 1 | 7.0 | 12 | 75.0 | 4 | 25.0 | 0 | 0.0 | 0 | 0.0 | 0 | 0.0 | 0 | 0.0 |  |
| Breastfed / fed baby | 7 | 88.0 | 1 | 12.0 | 7 | 70.0 | 3 | 30.0 | 5 | 63.0 | 3 | 37.0 | 4 | 57.0 | 3 | 43.0 | 3 | 30.0 | 7 | 70.0 | 9 | 69.0 | 4 | 31.0 |  |
| Cleaned playspace | 0 | 0.0 | 1 | 100.0 | 0 | 0.0 | 0 | 0.0 | 0 | 0.0 | 3 | 100.0 | 0 | 0.0 | 4 | 100.0 | 0 | 0.0 | 0 | 0.0 | 0 | 0.0 | 0 | 0.0 |  |
| Washed infant | 0 | 0 | 2 | 100.0 | 0 | 0.0 | 3 | 100.0 | 0 | 0.0 | 4 | 100.0 | 0 | 0.0 | 5 | 100.0 | 0 | 0.0 | 12 | 100.0 | 0 | 0.0 | 6 | 100.0 |  |
| Chopped wood | 0 | 0 | 0 | 0.0 | 0 | 0.0 | 0 | 0.0 | 6 | 86.0 | 1 | 14.0 | 6 | 75.0 | 2 | 25.0 | 0 | 0.0 | 0 | 0.0 | 0 | 0.0 | 0 | 0.0 |  |
| Visited neighbours/ other | 1 | 50 | 1 | 50.0 | 1 | 33.0 | 2 | 67.0 | 1 | 50.0 | 1 | 50.0 | 2 | 40.0 | 3 | 60.0 | 0 | 0.0 | 0 | 0.0 | 0 | 0.0 | 4 | 100.0 |  |
| Ate a meal | 0 | 0.0 | 0 | 0.0 | 0 | 0.0 | 0 | 0.0 | 0 | 0.0 | 0 | 0.0 | 0 | 0.0 | 2 | 100.0 | 0 | 0.0 | 0 | 0.0 | 6 | 26.0 | 17 | 74.0 |  |
| Slept / rested | 0 | 0.0 | 0 | 0.0 | 0 | 0.0 | 0 | 0.0 | 0 | 0.0 | 2 | 100.0 | 3 | 60.0 | 2 | 40.0 | 5 | 24.0 | 16 | 76.0 | 0 | 0.0 | 13 | 100.0 |  |
| HPS, household playspace.  ^*^Number represents reported incidence of that activity within the past 24 hours. Households (n=50) were asked an open-ended question on their daily activities during the past 24 hours. Not every activity was reported by every respondent.  ^**^Percentages are calculated from the total number of households who reported that activity. | | | | | | | | | | | | | | | | | | | | | | | | | |

**Table C. Reported playspace use and non-use during daily activities in the past 24 hours across daily time periods: at two and four weeks.**

|  | **Morning** | | **Afternoon** | | **Evening** | |
| --- | --- | --- | --- | --- | --- | --- |
|  | Two weeks | Four weeks | Two weeks | Four weeks | Two weeks | Four weeks |
| Reported use of HPS | 154 | 153 | 119 | 153 | 93 | 105 |
| Reported non-use of HPS | 24 | 32 | 28 | 32 | 42 | 60 |
| HPS, household playspace.  Figures are summed from reported daily activities table in S4. | | | | | | |

**Table D. Modified Barrier Analysis results among the study intervention group.**

| **BA determinant and question** | | Very… | | Quite… | | Not… | | Don’t know | |
| --- | --- | --- | --- | --- | --- | --- | --- | --- | --- |
|  |  | n  (50) | % | n  (50) | % | n  (50) | % | n  (50) | % |
| *Cues for action/ reminders* | How **difficult** is it to remember to use the HPS every time you could? | 0 | 0.0 | 0 | 0.0 | 50 | 100.0 | 0 | 0.0 |
| *Perceived susceptibility/ risk* | How **likely** do you think it is your child will get diarrhoea within the next month? | 0 | 0.0 | 9 | 18.0 | 24 | 48.0 | 17 | 34.0 |
| *Perceived severity* | How **serious** would it be if your child had diarrhoea? | 40 | 80.0 | 7 | 14.0 | 2 | 4.0 | 1 | 2.0 |
| *Perceived action efficacy* | How **likely** is it your child will get diarrhoea if you used the HPS whenever you could? | 0 | 0.0 | 9 | 18.0 | 38 | 76.0 | 3 | 6.0 |
|  | | Yes | | No | | Don’t know | |  | |
|  |  | n  (50) | % | n  (50) | % | n  (50) | % |  |  |
| *Perceived divine will* | Do you think God approves of you using the HPS? | 48 | 96.0 | 1 | 2.0 | 0 | 0.0 |  |  |
| *Policy* | Are there any community rules which prevent you from using the HPS? | 0 | 0.0 | 50 | 100.0 | 0 | 0.0 |  |  |
| *Culture* | Are there any cultural rules that you know of against using the HPS? | 0 | 0.0 | 50 | 100.0 | 0 | 0.0 |  |  |
| HPS, household playspace. | | | | | | | | | |

| **BA determinant** | **BA question** | **Inductive theme** | n  (50) | % |
| --- | --- | --- | --- | --- |
| *Perceived positive consequences* | What are the advantages of using the HPS? | Prevents ingestion of dirt/soil/dirty objects | 40 | 80.0 |
|  |  | Prevents injury (falling, fire, drowning, dust/ash, road) | 38 | 76.0 |
|  |  | Prevents injury from animals | 29 | 58.0 |
|  |  | Decreases/Eases mother's workload | 28 | 56.0 |
|  |  | Mother worries less for infant's health/safety | 26 | 52.0 |
|  |  | Eases time pressure for mother/stress | 23 | 46.0 |
|  |  | Improves infant’s physical development | 21 | 42.0 |
|  |  | Infant/Clothes stay clean | 20 | 40.0 |
|  |  | Prevents ingestion of faeces | 20 | 40.0 |
|  |  | Infant feels happy playing inside/comfortable | 18 | 36.0 |
|  |  | Prevents diarrhoea/Other disease | 14 | 28.0 |
|  |  | Protects from sunlight | 4 | 8.0 |
|  |  | Promotes infant's independence | 2 | 4.0 |
| *Perceived negative consequences* | What are the disadvantages of using the HPS? | No disadvantage | 26 | 52.0 |
|  |  | Cost of extra cleaning materials | 11 | 22.0 |
|  |  | Takes up space inside the home | 7 | 14.0 |
|  |  | Infant cries (from boredom) | 7 | 14.0 |
|  |  | Extra item to clean | 6 | 12.0 |
| *Perceived self-efficacy* | What makes it easy for you to use the HPS? | Easy to assemble/rope easy to tie | 27 | 54.0 |
|  |  | Weighs little/Easy to move (including mattress) | 25 | 50.0 |
|  |  | Good size/Takes little space inside | 22 | 44.0 |
|  |  | Door facilitates easy use | 14 | 28.0 |
|  |  | Safe design/Infant easily visible | 14 | 28.0 |
|  |  | Design encourages infant play (size/comfort) | 13 | 26.0 |
|  |  | Bamboo structure strong/Stable/Durable | 12 | 24.0 |
|  |  | Can be taken outside | 8 | 16.0 |
|  |  | Older children who can watch infant | 8 | 16.0 |
|  |  | Good width to slats to encourage standing | 4 | 8.0 |
|  | What makes it difficult for you to use the HPS? | Difficult to rethread rope when dismantled | 19 | 38.0 |
|  |  | No older children to watch infant | 16 | 32.0 |
|  |  | Lack of toys | 16 | 32.0 |
|  |  | Difficult to move outside/Heavy without help | 8 | 16.0 |
|  |  | Rope may become loose/Structure falls | 4 | 8.0 |
|  |  | Nothing | 3 | 6.0 |
|  |  | Takes up space/House is small | 2 | 4.0 |
|  |  | No older children to watch infant | 2 | 4.0 |
|  |  | Plastic can get hot in sun | 1 | 2.0 |
|  |  | Height insufficient | 1 | 2.0 |
| *Access* | What makes it easy for you to keep the HPS clean? | Plastic covering easily cleaned | 39 | 78.0 |
|  |  | Mattress lightweight/Small to carry/Removable | 38 | 76.0 |
|  |  | Requires little water | 34 | 68.0 |
|  |  | Dries quickly (in/out of sun) | 16 | 32.0 |
|  |  | Bamboo stays clean/easy to wipe | 14 | 28.0 |
|  |  | Requires little soap | 13 | 26.0 |
|  |  | Plastic does not absorb smell/urine/dirt | 5 | 10.0 |
|  |  | Soap easy to buy/Inexpensive | 2 | 4.0 |
|  |  | Water easily available | 2 | 4.0 |
|  | What makes it difficult for you to keep the HPS clean? | Lack of/Expense of buying soap | 28 | 56.0 |
|  |  | Water unavailable at times | 13 | 26.0 |
|  |  | Requires extra cleaning materials/Associated cost | 12 | 24.0 |
|  |  | Nothing | 6 | 12.0 |
|  |  | Material (rough bamboo/rope/open seams) | 5 | 10.0 |
| *Perceived social norms* | Who are the people who approve of you using the HPS? | Neighbours | 48 | 96.0 |
|  |  | Immediate family (parents, grandparents, siblings) | 33 | 66.0 |
|  |  | HEW/HDA/Other government worker | 24 | 48.0 |
|  |  | Husband | 20 | 40.0 |
|  |  | Friends of parents | 18 | 36.0 |
|  |  | Aunts/Uncles/Family-in-law | 18 | 36.0 |
|  |  | Community members/Guests/Passers-by | 11 | 22.0 |
|  |  | Labourer/Customers | 3 | 6.0 |
|  | Who are the people who disapprove of you using the HPS? | Nobody | 37 | 74.0 |
|  |  | Friends of parents | 6 | 12.0 |
|  |  | Neighbours | 4 | 8.0 |
|  |  | Community members/Colleagues/Customers | 3 | 6.0 |
| BA, Barrier Analysis; HPS, household playspace. | | | | |

**Table E. Number of samples positive for presumptive *Campylobacter* spp. under each category of colony count.**

|  | Intervention  (n=50) | | | | | Control  (n=50) | | | | |
| --- | --- | --- | --- | --- | --- | --- | --- | --- | --- | --- |
|  | 0-100 | 101-250 | 251-999 | >1000 | >2000 | 0-100 | 101-250 | 251-999 | >1000 | >2000 |
| Baseline | 2 | 2 | 14 | 6 | 3 | 3 | 10 | 13 | 0 | 0 |
| Two weeks | 0 | 1 | 14 | 2 | 0 | 4 | 4 | 8 | 1 | 1 |
| Four weeks | 2 | 6 | 4 | 2 | 0 | 3 | 5 | 6 | 0 | 0 |

**Table F. Feeding of fresh or reheated foods prepared as recommended, across study groups and time points.**

| **Baseline** | | | | | | | |
| --- | --- | --- | --- | --- | --- | --- | --- |
|  | Intervention (n=50) | | Control (n=50) | | Total (N=100) | |  |
|  | n | % | n | % | n | % |  |
| Morning meal | 30 | 60.0 | 43 | 86.0 | 73 | 73.0 |  |
| Midday meal | 34 | 68.0 | 38 | 76.0 | 72 | 72.0 |  |
| Evening meal | 33 | 66.0 | 42 | 84.0 | 75 | 75.0 |  |
| All safely prepared | 25 | 50.0 | 31 | 62.0 | 56 | 56.0 |  |
| **Two weeks** | | | | | | | |
|  | Intervention (n=50) | | Control (n=50) | | Total (N=100) | |  |
|  | n | % | n | % | n | % |  |
| Morning meal | 31 | 62.0 | 38 | 76.0 | 69 | 69.0 |  |
| Midday meal | 41 | 82.0 | 38 | 76.0 | 79 | 79.0 |  |
| Evening meal | 37 | 74.0 | 39 | 78.0 | 76 | 76.0 |  |
| All safely prepared | 19 | 38.0 | 28 | 56.0 | 47 | 47.0 |  |
| **Four weeks** | | | | | | | |
|  | Intervention (n=50) | | Control (n=50) | | Total (N=100) | |  |
|  | n | % | n | % | n | % |  |
| Morning meal | 37 | 74.0 | 36 | 72.0 | 73 | 73.0 |  |
| Midday meal | 30 | 60.0 | 30 | 60.0 | 60 | 60.0 |  |
| Evening meal | 31 | 62.0 | 37 | 74.0 | 68 | 68.0 |  |
| All safely prepared | 21 | 42.0 | 24 | 48.0 | 45 | 45.0 |  |
| Feeding of fresh or reheated foods ’prepared as recommended’ if: Meals were prepared less than 2 hours before eating (not reheated); Meals were prepared less than 2 hours before eating which were reheated to ‘almost boiling’; Meals were prepared more than 2 hours before eating but reheated to ‘almost boiling’.  ‘Not prepared as recommended’ if: Meals were prepared less than 2 hours before eating but only heated to ‘warm’; Meals were prepared more than 2 hours before eating and reheated to ‘warm’. | | | | | | | |

**Table G Number of meals safely prepared across time points**

|  | All meals safely prepared at 14 days  (N=100) | All meals safely prepared at four weeks  (N=100) | All meals safely prepared from baseline  (N=100)* |
| --- | --- | --- | --- |
| Intervention and control households | 47 | 45 | 28 |
| *Calculated as the sum of households who safely prepared their food at both time points, as defined in table S8 footnotes. | | | |
