# Supplementary material for: A randomised controlled feasibility trial of a BabyWASH household playspace: The CAMPI study
Source: PLoS Negl Trop Dis. 2021 Jul 14;15(7):e0009514. doi: 10.1371/journal.pntd.0009514 (PMC8312948; doi:10.1371/journal.pntd.0009514)
Supplement: Attachment — Submitted filename: PNTD-D-20-01355_reviewer.pdf [file pntd.0009514.s005.pdf]

PLOS Neglected Tropical Diseases

A randomised controlled feasibility trial of a BabyWASH household playspace: The CAMPI study

--Manuscript Draft--

**Manuscript Number:**	PNTD-D-20-01355
**Full Title:**	A randomised controlled feasibility trial of a BabyWASH household playspace: The CAMPI study
**Short Title:**	A randomised controlled feasibility trial of a household playspace
**Article Type:**	Research Article
**Keywords:**	BabyWASH; Campylobacter; hygiene; infant health; infection; playspace; sanitation; WASH.
**Abstract:**	Background. Water, sanitation and hygiene (WASH) interventions should support infant growth but trial results are inconsistent. Frequently, interventions do not consider behaviours or transmission pathways specific to age. A household playspace (HPS) is one intervention component which may block faecal-oral transmission. This study was a two-armed, parallel-group, randomised, controlled feasibility trial of a HPS in rural Ethiopia. It aimed to recommend proceeding to a definitive trial. Secondary outcomes included effects on infant health, injury prevention and women's time. Methods. November 2019−January 2020 106 households were identified and assessed for eligibility. Recruited households (N=100) were randomised (blinded) to intervention or control (both n=50). Outcomes included recruitment, attrition, adherence, and acceptability. Data were collected at baseline, two and four weeks. Findings. Recruitment met a priori criteria (≥80%). There was no loss to follow-up, and no non-use, meeting adherence criteria (both ≤10%). Further, 48.0% (95% CI 33·7−62·6; n=24) of households appropriately used and 56·0% (41·3−70·0; n=28) cleaned the HPS over four weeks, partly meeting adherence criteria (≥50%). For acceptability, 41·0% (31·3−51·3; n=41) of infants were in the HPS during random visits, failing criteria (≥50%). Further, the proportion of HPS use decreased during some activities, failing criteria (no decrease in use). A modified Barrier Analysis described good acceptability and multiple secondary benefits, including on women's time burden and infant injury prevention. Interpretation. Despite failing some a priori criteria, the trial demonstrated good feasibility among intervention households. A definitive trial to determine efficacy is warranted. Funding. People In Need; Czech Development Agency. Trial registration. RIDIE-ID-5de0b6938afb8.
**Additional Information:**	
**Question**	**Response**
**Financial Disclosure**<br><br>Enter a financial disclosure statement that describes the sources of funding for the work included in this submission. Review the submission guidelines for detailed requirements. View published research articles from *PLOS NTDs* for specific examples.<br><br>This statement is required for submission and **will appear in the published article** if the submission is accepted. Please make sure it is accurate.	SB is jointly funded as a research student by both Cranfield University and People In Need, who received funding from the Czech Development Agency for the project. No other external funds supported this work.

Powered by Editorial Manager® and ProduXion Manager® from Aries Systems Corporation

**Unfunded studies**<br><br>Enter: *The author(s) received no specific funding for this work.*<br><br>**Funded studies**<br><br>Enter a statement with the following details:<br>• Initials of the authors who received each award<br>• Grant numbers awarded to each author<br>• The full name of each funder<br>• URL of each funder website<br>• Did the sponsors or funders play any role in the study design, data collection and analysis, decision to publish, or preparation of the manuscript?<br>• **NO** - Include this sentence at the end of your statement: *The funders had no role in study design, data collection and analysis, decision to publish, or preparation of the manuscript.*<br>• **YES** - Specify the role(s) played.<br><br>* typeset	
**Competing Interests**<br><br>Use the instructions below to enter a competing interest statement for this submission. On behalf of all authors, disclose any competing interests that could be perceived to bias this work—acknowledging all financial support and any other relevant financial or non-financial competing interests.<br><br>This statement **will appear in the published article** if the submission is accepted. Please make sure it is accurate. View published research articles from *PLOS NTDs* for specific examples.	The authors have declared that no competing interests exist.

**NO authors have competing interests**<br><br>Enter: *The authors have declared that no competing interests exist.*<br><br>**Authors with competing interests**<br><br>Enter competing interest details beginning with this statement:<br><br>*I have read the journal's policy and the authors of this manuscript have the following competing interests: [insert competing interests here]*<br><br>* typeset	
**Data Availability**<br><br>Authors are required to make all data underlying the findings described fully available, without restriction, and from the time of publication. PLOS allows rare exceptions to address legal and ethical concerns. See the PLOS Data Policy and FAQ for detailed information.<br><br>A Data Availability Statement describing where the data can be found is required at submission. Your answers to this question constitute the Data Availability Statement and **will be published in the article**, if accepted.	No - some restrictions will apply

**Important:** Stating 'data available on request from the author' is not sufficient. If your data are only available upon request, select 'No' for the first question and explain your exceptional situation in the text box.<br><br>Do the authors confirm that all data underlying the findings described in their manuscript are fully available without restriction?	
**Describe where the data may be found in full sentences. If you are copying our sample text, replace any instances of XXX with the appropriate details.**<br><br>• If the data are **held or will be held in a public repository**, include URLs, accession numbers or DOIs. If this information will only be available after acceptance, indicate this by ticking the box below. For example: *All XXX files are available from the XXX database (accession number(s) XXX, XXX.*).<br>• If the data are all contained **within the manuscript and/or Supporting Information files**, enter the following: *All relevant data are within the manuscript and its Supporting Information files.*<br>• If neither of these applies but you are able to provide **details of access elsewhere**, with or without limitations, please do so. For example:<br><br>*Data cannot be shared publicly because of [XXX]. Data are available from the XXX Institutional Data Access / Ethics Committee (contact via XXX) for researchers who meet the criteria for access to confidential data.*<br><br>*The data underlying the results presented in the study are available*	Data will be made publically available on free repositories upon publication.

Powered by Editorial Manager® and ProduXion Manager® from Aries Systems Corporation

from (include the name of the third party and contact information or URL).<br>• This text is appropriate if the data are owned by a third party and authors do not have permission to share the data.<br><br>* typeset	
Additional data availability information:	

Powered by Editorial Manager® and ProduXion Manager® from Aries Systems Corporation

Cover Letter
Click here to access/download;Manuscript;S Budge et al 2020_Manuscript.docx

Full title

A randomised controlled feasibility trial of a BabyWASH household playspace: The CAMPI study

Short title

A randomised controlled feasibility trial of a household playspace

Author names

Sophie Budge[a¶], Paul Hutchings[b¶*], Alison Parker[a¶], Sean Tyrrel[a¶], Sam Norton[c¶], Camila Garbutt[d&], Fitsume Woldemedhin[e&], MohammedYasin Jemal[e&], Mathewos Moges[f&], Siraj Hussen Adem[f&], Hunachew Beyene[f&]

Author affiliations

[a]*Cranfield Water Science Institute, Cranfield University, Cranfield, UK.*

[b] *Faculty of Engineering and Physical Sciences, University of Leeds, Leeds, UK*

[c]*Psychology Department, Institute of Psychiatry Psychology & Neuroscience, King's College London, London, UK.*

[d]*PIN UK, East Road, London, UK.*

[e]*PIN, Hawassa, Ethiopia.*

[f]*Hawassa University College of Medicine and Health Sciences, Hawassa, Ethiopia.*

[*]Corresponding author.

Email address: p.hutchings@leeds.ac.uk (PT)

¶ These authors contributed equally to this work.

& These authors also contributed equally to this work.

Abstract

Background. Water, sanitation and hygiene (WASH) interventions should support infant growth but trial results are inconsistent. Frequently, interventions do not consider behaviours or transmission pathways specific to age. A household playspace (HPS) is one intervention component which may block faecal-oral transmission. This study was a two-armed, parallel-group, randomised, controlled feasibility trial of a HPS in rural Ethiopia. It aimed to recommend proceeding to a definitive trial. Secondary outcomes included effects on infant health, injury prevention and women's time. **Methods.** November 2019−January 2020 106 households were identified and assessed for eligibility. Recruited households (N=100) were randomised (blinded) to intervention or control (both n=50). Outcomes included recruitment, attrition, adherence, and acceptability. Data were collected at baseline, two and four weeks. **Findings.** Recruitment met *a priori* criteria (≥80%). There was no loss to follow-up, and no non-use, meeting adherence criteria (both ≤10%). Further, 48.0% (95% CI 33·7−62·6; n=24) of households appropriately used and 56·0% (41·3−70·0; n=28) cleaned the HPS over four weeks, partly meeting adherence criteria (≥50%). For acceptability, 41·0% (31·3−51·3; n=41) of infants were in the HPS during random visits, failing criteria (≥50%). Further, the proportion of HPS use decreased during some activities, failing criteria (no decrease in use). A modified Barrier Analysis described good acceptability and multiple secondary benefits, including on women's time burden and infant injury prevention. **Interpretation.** Despite failing some *a priori* criteria, the trial demonstrated good feasibility among intervention households. A definitive trial to determine efficacy is warranted.
Funding. People In Need; Czech Development Agency. **Trial registration.** RIDIE-ID-5de0b6938afb8.

Keywords

BabyWASH, Campylobacter, hygiene, infant health, infection, playspace, sanitation, WASH.

Background

Final height in adults results from many environmental factors which support growth in childhood.[1] Conversely, adverse influences which begin *in utero* and continue through puberty can lead to growth failure.[1] This includes the cyclical relationship between infection and nutrition. Symptomatic infection is common during early years in low-income countries, and repeated diarrhoea impairs growth, weight gain and long-term cognitive development.[2] Moreover, enteric infections which are asymptomatic but which result in subclinical enteropathy[3] are also associated with growth shortfalls[4,5] – suggesting infection affects development without overt outcomes like diarrhoea. Population-level nutrition and hygiene status are thus critical for proper growth, but are not sufficient alone: where there are widespread infection and inflammation, the effect of nutrition on growth is seriously compromised.[1] Indeed, the modest effects on growth in nutrition interventions suggests that a combination of recurrent infections, chronic inflammation, and gut enteropathy limit the effects of nutrition.[6] Thus randomised controlled trials (RCT) are testing water, sanitation and hygiene (WASH) interventions alongside supplementary nutrition to improve infant health.

Despite substantial evidence suggesting safe WASH contributes to good child health,[7] RCTs testing improved household WASH (with or without supplementary nutrition) have shown variable, mostly insignificant, effects.[8–11] Whilst it is improbable that interventions at the coverage in these trials will alleviate growth failure, results have prompted discourse on what is necessary. The concept 'Transformative WASH'[12] highlights the necessity of substantially improving environmental hygiene amongst the poorest, whom disproportionately experience poor child health. It also recognises the significant burden of contamination from domestic animals – largely unaddressed in WASH trials or programs.[13] In rural, subsistence agriculture settings it is common for domestic animals to share living and sleeping spaces. Acting as natural reservoirs for several zoonotic pathogens, they likely contribute substantial contamination to multiple transmission routes,[14,15] associated with growth failure and gut abnormalities.[16,17] Further, a transformative approach will require that interventions (whether technical, structural, or behavioural) consider age-related behaviours and transmission pathways to

prevent infant infection.[18] One such 'critical' WASH intervention component[19] is a household playspace (HPS) which might help reduce faecal-oral transmission from ingested soil and faeces,[20,21] and contaminated floors.[22] In rural areas, a HPS may offer some protection from infection during early growth periods.

The evidence on the health and non-health benefits of a HPS or playmat has been previously reviewed.[23] Further, formative data during the participatory design and build of the HPS prototype suggested caregivers liked it and were glad to use it during daily routines. However, there remains a need to assess how long a HPS would be used throughout the day and appropriately maintained and cleaned. Data on infant health outcomes would provide insight into the potential for a HPS to reduce infection from within the home. Moreover, WASH interventions deliver both health and non-health outcomes, all of which contribute to household wellbeing. Thus broader benefits of a HPS, including on women's' time and child socioemotional development, also require exploration through a definitive RCT.

Aims

The Campylobacter-Associated Malnutrition Playspace Intervention (CAMPI) trial was a randomised, controlled feasibility trial to establish the feasibility of a definitive RCT of a HPS in rural Ethiopia. The HPS design (S1), is described elsewhere,[23] underpinned by previous formative research.[24–26]

Primary aims of the trial were to:

1. Establish the feasibility of future definitive RCT to evaluate efficacy of a HPS
2. Evaluate the HPS as a public health intervention through measures of recruitment, attrition, adherence, and acceptability, and as efficacy methods within a RCT
3. Evaluate the appropriateness of the study design and make recommendations to adjust the intervention and design for future trials.

Too many objectives, there must be a primary objective and secondary objectives.
Perhaps divide your objectives into operational and health objectives.
You would have enough data to produce 2 complementary publications

As formal hypothesis testing for effectiveness is not recommended in feasibility studies, the trial did not aim to determine the effect of the HPS on health outcomes, and was not powered for this. However, further evidence was required towards the infection-exposure hypothesis as well as effects on broader outcomes. Thus secondary outcomes aimed to:

1. Confirm the prevalence of *Campylobacter* infection in the study population
2. Describe effects of the HPS on *Campylobacter* infection and diarrhoea
3. Describe secondary effects, including on women's use of time, childcare, or injury prevention.

Methods

Design

This feasibility trial was designed by Cranfield University alongside People in Need (PIN) and Hawassa University and conducted in the Southern Nations, Nationalities and Peoples' region, Ethiopia. It was a two-armed, parallel-group, randomised controlled feasibility trial with equal group allocation. The Consolidated Standards of Reporting Trials (CONSORT) 2010 statement with extension to pilot trials was followed during study design and reporting.

Randomisation and masking

As a feasibility trial, a sample size calculation was not performed. A target of 100 households was deemed sufficient to inform researchers about practicalities of running the trial and for sufficient precision to estimate rates of recruitment, retention, and trial outcomes. Eligible households were identified, contacted and enrolled into the trial November 2019−January 2020. Four kebeles (two intervention, two control) were chosen from a woreda (zonal subdivision) representative of rural livelihoods across the region, without geographical overlap. Alongside government Health Extension Workers (HEW), PIN team members produced a blinded sampling frame from kebeles of all households fulfilling eligibility criteria. Households were sequentially numbered and using statistical software, 25 households were randomly drawn from each frame for a total sample of 100 (50 intervention, 50 control). Inclusion criteria were: 1. Subsistence agriculture households raising

domestic animals, within PIN intervention scope; 2. With an infant aged 8−16 months (10−18 months at trial commencement); 3. Not participating in other PIN projects. Exclusion criteria: 1. Outside 10−18 month range at trial start; 2. Participating in other PIN projects; 3. Infant was pre-term, low birth weight, or had other birth complications. PIN staff and HEWs approached households with the study information and participants were given time to make an informed decision. Households were then revisited, eligibility was re-verified, and if households were willing, consent was gained. Households were blinded to their status in the trial until after baseline data collection. Figure 1 describes trial enrollment and numbers.

[Insert]

Figure 1. Modified version of CONSORT 2010 flow diagram of participants in the CAMPI feasibility trial.

Study intervention

Country

The trial was conducted in Sidama zone, January−March 2020. Two field teams managed intervention and control kebeles. After baseline data collection, caregivers from intervention households were called to the kebele health post for a ‘sensitisation’ day. PIN field team, HEWs and data collectors formally discussed the study rationale, caregiver beliefs around infant faecal-oral transmission and health outcomes, transmission routes and how a HPS might interrupt these to improve infant health. Correct HPS use, maintenance and cleaning was detailed. Caregivers watched and practiced HPS assembly and discussed potential safety issues. Use was discussed in relation to daily routines and activities and caregivers agreed to use it when possible. Households agreed to clean the HPS at least every other day (and always after defecation or urination) with both soap and water. Playspaces were distributed with safety instructions printed in both Sidamo and Amharic with illustrations. HEWs visited intervention households in the following days to ensure correct HPS assembly. The control group received a HPS upon study completion.

Participant data

Survey and anthropometry

Households were visited at baseline and at two and four weeks. The primary caregiver present was interviewed, usually the mother. Baseline data included a previously validated survey[25,26] on WASH facilities and use and animal husbandry. Food hygiene, breastfeeding, and diarrhoea incidence were also assessed and again at two and four weeks. Trained data collectors took weight, height and mid-upper arm circumference (MUAC) following standard procedure[27] with a digital mother-child smart scale (Ultratec®), a foldable infantometer to 5 mm accuracy (seca 210®) and standard MUAC tape to 1 mm accuracy, respectively. Seven day diarrhoea prevalence was by caregiver report.

Laboratory confirmation

Collection and processing of infant faecal samples followed a validated methodology.[26] Briefly, a day prior to household visits data collectors distributed sterile sample collection bags (Whirl-Pak®, Sigma-Aldrich, UK) and demonstrated sterile sample collection. After collection, samples were transported on ice for the isolation of presumptive *Campylobacter* spp. using CHROMagar™ selective media and appropriate microaerophilic conditions by trained laboratory staff at Hawassa University College of Medicine and Life Sciences. Samples were processed for all 100 households at each study time point.

Implementation outcomes

Evaluation of trial outcomes and proceeding with future definitive trial

Among the intervention group, surveys at two and four weeks assessed feasibility outcomes: Recruitment (number of households contacted who consented); Attrition (the proportion of participants lost to follow-up at the trial end); Adherence (proportion of HPS non-use, as well as Appropriate use/maintenance and cleaning), and Acceptability (random observation of HPS use and change in incidence [proportion] of use from two to four weeks). A modified Barrier Analysis at four weeks provided further insight into acceptability. As these outcomes were the main measures to determine whether to proceed to a definitive trial, *a priori* threshold criteria were established as follows: 1. Recruitment: the proportion of contacted households participating in the trial would be ≥80%; 2. Attrition: the level at the trial end would be ≤10%; 3. Adherence: the proportion of non-use

of HPS would be ≤10% at both time points and over the trial; 4. Adherence: the proportion of correct HPS use and cleaning would be ≥50% at both time points and over the trial; 5. Acceptability: the proportion of infants in the HPS at random check would be ≥50% at both time points and over the trial, and 6. Acceptability: reported incidence of HPS use during daily activities (as a proportion) would not decrease from two to four weeks. Outcomes would also indicate appropriateness of an RCT and provide recommendations for adjusting the intervention design.

Statistical analysis

Data were managed in Excel and analysed in SPSS (v26, IBM). Descriptive statistics summarised survey data and health outcomes. Trial outcomes are displayed with 95% confidence intervals (CI). The adherence outcome included 'Appropriate use' and 'Appropriate cleaning', created as composite binary outcome variables (described in table footnotes) and described across study time points. Adherence as 'HPS non-use' was described as reported non-use after baseline. Acceptability as 'Infant in playspace upon arrival' was calculated for both visits. Acceptability as change in HPS use was calculated from reported HPS use during reported daily activities over two and four weeks and the difference in proportions. A Generalised Estimating Equaton (GEE) was used as a semi-parametric model, using a robust variance estimator and an unstructured working correlation matrix. A binary logistic GEE estimated factors associated with 'Appropriate use' and 'Appropriate cleaning' at two and four weeks. Models were initially run separately: however the merged composite variable of 'Appropriate use and cleaning' showed no difference in parameter estimates between models and is presented. Pre-specified variables included infant sex and age; maternal age; maternal education; number in household; number of children; household owns soap; safe water storage; animal husbandry practices; water availability, and mother collects water. Results are expressed as populated averaged odds ratios (ORs) with 95% CI.

Acceptability was further assessed through a modified Barrier Analysis which explored determinants of use among all participants. Methods and analysis are described in detail in supplementary information (S1). Derivation of themes was data-driven, where codes resulted from the analysed data

as they related to each determinant (See S1 and S6). Coded themes are discussed as either barriers or enablers to the implantation of, and improving outcomes during, a definitive trial. For secondary health outcomes, anthropometric z-scores were calculated (WHO Anthro v3·2·2) and categorised into stunting and wasting using standard cut-off values.[28] Samples positive for presumptive *Campylobacter* spp., colonies were counted using OpenCFU. Change in diarrhoeal and *Campylobacter* prevalence between study groups was estimated using a GEE intercept-only model with OR and 95% CI.

Ethics

Upon recruitment, PIN staff and HEWs discussed the study with primary caregivers who understood data were anonymous. Informed consent and assent on behalf of infant participants was obtained, or thumbprints taken. Surveys were translated to Amharic by PIN staff and administered verbally in Amharic or Sidamo. Various checks throughout the trial assessed HPS safety and monitored for adverse events. This included regular survey checkpoints (data concerns from households and HPS safety and visual inspection of HPS for unsafe use or assembly), and the distribution of feedback response mechanism cards to contact PIN staff. Infants with moderate or severe acute malnutrition measured by MUAC were advised to contact their local health post, which was followed up by a HEW. The study was approved by Cranfield University Research Ethics Committee (CURES/9357/2019) and Hawassa University College of Medicine and Health Sciences Institutional Review Board (IRB/010/12).

Results

Baseline characteristics

Household demographic characteristics are described in Table 1 for both study groups and as a whole. Characteristics were largely balanced across groups. Average infant age was 10·8 months (median 10·0; range 7−18). Average length-for-age (LAZ) and weight-for-length (WLZ) at baseline did not vary substantially across intervention and control groups at -1·00 and -0·96 (LAZ) and -0·49 and -

0·46 (WLZ) respectively. Stunting and wasting affected 33·0% (n=33) and 13·0% (n=13) of all infants respectively with some severe acute malnutrition (11·0%, n=11). Mothers were mostly aged 18−25 (50·0%, n=25; 62·0%, n=31, respectively) and educated to second grade (44·0%, n=22; 52·0%, n=26, respectively). Whilst most households had a pit latrine with a slab (51·0%, n=51), open defecation was still common (19·0%, n=19). Cattle and chickens were the most frequent domestic animal, and husbandry practices indicated animals frequently shared living spaces during the day and night, with infrequent use of pens.

	Intervention (n=50)		Control (n=50)		Total (N=100)	
	n	%	n	%	n	%
**Demographics**						
Infant sex: Male	28	56·0	24	48·0	52	52·0
Average infant age (months)	10·1		11·6		10·8	
Respondent: Mother	45	90·0	48	96·0	93	93·0
Maternal age: <18	1	2·0	0	0·0	1	1·0
18-25	25	50·0	31	62·0	56	56·0
26-35	21	42·0	18	36·0	39	39·0
36-45	3	6·0	0	0·0	3	3·0
>45	0	0·0	1	2·0	1	1·0
Maternal education: Cannot read/write	12	24·0	6	12·0	18	18·0
First grade	13	26·0	15	30·0	28	28·0
Second grade	22	44·0	26	52·0	48	48·0
Secondary and above	3	6·0	3	6·0	6	6·0
Number in household: 1-3	5	10·0	9	18·0	14	14·0
4-6	34	68·0	33	66·0	67	67·0
7+	11	22·0	8	16·0	19	19·0
Number of children: 1-2	22	44·0	23	46·0	45	45·0
3-4	18	36·0	23	46·0	41	41·0
5-6	9	18·0	4	8·0	13	13·0
7+	1	2·0	0	0·0	1	1·0
Number of children ≤5: 1	35	70·0	36	72·0	71	71·0
2	12	24·0	13	26·0	25	25·0
3	3	6·0	1	2·0	4	4·0
Main income: Farming/livestock	48	96·0	49	98·0	97	97·0
Trade	17	34·0	19	38·0	36	36·0
Employee	1	2·0	0	0·0	1	1·0
Household has formal means of saving	9	18·0	8	16·0	17	17·0
House material: Wood and mud	38	76·0	47	94·0	85	85·0
Wood and grass	10	20·0	1	2·0	11	11·0
Concrete	2	4·0	2	4·0	4	4·0
Floor material: Concrete / cement	16	32·0	9	18·0	25	25·0
Mud / soil	34	68·0	41	82·0	75	75·0
	**Intervention** (n=50)		**Control** (n=50)		**Total** (N=100)	
	n	%	n	%	n	%
**WASH indicators**						
Latrine type: Defecate in open	8	16·0	11	5·0	19	19·0
Share neighbour's	6	12·0	8	16·0	14	14·0
Pit latrine without slab	11	22·0	5	10·0	16	16·0
Pit latrine with slab	25	50·0	26	52·0	51	51·0
Water source: Piped water / public tap	50	50·0	50	50·0	100	100·0
Who collects water?: Mother	39	78·0	39	78·0	78	100·0
Father	8	16·0	3	6·0	11	11·0
A grandparent	0	0·0	1	2·0	1	1·0
Female child (≤15)	11	22·0	10	20·0	21	21·0
Male child (≤15)	3	6·0	7	14·0	10	10·0
Labourer	1	2·0	11	22·0	12	12·0
Water available inside the home	43	86·0	46	92·0	89	89·0
Household safely stores water*	12	24·0	14	28·0	26	26·0
Household owns soap	34	68·0	39	78·0	73	100·0
**Animal husbandry**						
Number of cattle: 1-3	31	62·0	25	25·0	56	56·0
4-6	9	18·0	11	22·0	20	20·0
7+	0	0·0	1	2·0	1	1·0

Number of goats: 1-3	8	16·0	8	16·0	16	16·0
4-6	2	4·0	1	2·0	3	3·0
7+	1	2·0	1	2·0	2	2·0
Number of donkey: 1-3	1	2·0	6	12·0	7	7·0
Number of sheep: 1-3	0	0·0	10	20·0	10	10·0
Number of chickens: 1-3	11	22·0	16	32·0	27	27·0
4-6	15	30·0	16	32·0	31	31·0
7+	18	36·0	7	14·0	25	25·0
Animal dwelling during the day<br>Outside, enclosed in a pen	1	2·0	0	0·0	1	1·0
Outside, roaming free	49	98·0	48	96·0	97	97·0
Inside, same room as family	33	66·0	40	80·0	73	73·0
Inside, separate room	1	2·0	2	4·0	3	3·0
Animal dwelling during the night<br>Outside, enclosed in a pen	7	14·0	10	20·0	17	17·0
Outside, roaming free	0	0·0	0	0·0	0	0·0
Inside, same room as family	34	68·0	35	70·0	69	69·0
Inside, separate room	9	18·0	5	10·0	14	14·0
	**Intervention** (n=50)		**Control** (n=50)		**Total** (N=100)	
	n	%	n	%	n	%
**Nutrition indicators**						
LAZ z-score (average)	-1·00		-0·96		-0·98	
LAZ (range)	-3·04−0·80		-2·76−0·66		-3·04−0·80	
WLZ z-score (average)	-0·49		-0·46		-0·47	
WLZ (range)	-2·30−0·87		-2·41−0·75		-2·41−0·87	
MUAC[a] (mm; average)	138·2		138·1		138·2	
Stunting (LAZ ≤ −2 SD)	17	34·0	16	32·0	33	33·0
Wasting (WLZ ≤ −2 SD)	6	12·0	7	14·0	13	13·0
MUAC[a]: ≥135	36	72·0	39	78·0	75	75·0
125−135	8	16·0	6	12·0	14	13·0
115−124	6	12·0	5	10·0	11	11·0
≤115	0	0·0	0	0·0	0	0·0

WASH, water, sanitation and hygiene; LAZ, length-for-age; WLZ, weight-for-length; MUAC, mid-upper arm circumference, where: ≥135, no risk of undernutrition; 12·5-13·5, at risk of moderate acute undernutrition; 11·5-12·4, moderate acute undernutrition; ≤11·5, severe acute undernutrition.
[*]Calculated as households who were marked 'Yes' to all three observation-based questions: Are water containers clean; Do the water containers have a protecting cover; Does the container have a tap or narrow mouth for drawing the water.

Table 1. Household demographic characteristics, and water, sanitation and hygiene, animal husbandry and nutrition indicators across study groups and as a total at baseline (N=100).

Trial outcomes

For ease of assessment, study outcomes are described together in Table 2 and individually in sections below.

**Quantitative trial outcomes**										
**Outcome**	Definition / Indicator	*A priori* criteria	Proportion (N=50)							
			Baseline		Two weeks		Four weeks		Study duration	
			%	95% CI	%	95% CI	%	95% CI	%	95% CI
**Recruitment**	Proportion of contacted houses who consented	≥80%	94·3	88·1–97·9	-	-	-	-	-	-
**Attrition**	Loss to follow-up	≤10%	-	-	-	-	-	-	0·0	0·0–3·6
**Adherence**	Non-use of HPS	≤10%	-	-	0·0	0·0–0·07	0·0	0·0–0·07	0·0	0·0–0·07
	Appropriate use	≥50%	-	-	70·0	55·5–82·1	64·0	49·2–77·1	48·0	33·7–62·6
	Appropriate cleaning	≥50%	-	-	72·0	57·5–83·8	70·0	55·4–82·1	56·0	41·3–70·0
	Appropriate use and cleaning	≥50%	-	-	52·0	37·4–66·3	48·0	33·7–62·6	26·0	14·6–40·3
**Acceptability**	Infant in HPS upon arrival	≥50%	-	-	32·0	19·5–46·7	50·0	35·5–64·5	41·0	31·3–51·3
	Proportion of HPS use during daily activities	No decrease	-	-	-	-	-	-	Decrease during certain activities	

Table 2. Outcomes for the CAMPI trial to determine progression to a future definitive RCT, at two and four weeks and across the trial duration.

Recruitment and attrition

Rates for recruitment and attrition are shown in Table 2. One hundred households were recruited from four kebeles. To achieve this, 106 households were assessed for eligibility; four households were then excluded for not meeting infant age criteria at the study start and a further two did not consent to participate (Figure 1). Thus a recruitment rate of 94·3% (95% CI 88·1−97·9) met *a priori* criteria of ≥80%. All households completed the trial assessments at four weeks and there was no loss to follow-up (0·0%; 95% CI 0·0−3·6), meeting criteria for attrition (≤10% at trial end).

Adherence

Adherence was first described as the proportion of HPS non-use at both time points and over the study period (Table 2). No households reported not using the HPS at either time point or over the study duration (0.0%, 95% CI 0·0−0·71), meeting *a priori* criteria ≤10%. Second, adherence was described through 'Appropriate use' and 'Appropriate cleaning' and combined, across the study time points and throughout the trial (Table 3). Appropriate use included maintenance, as described in the table footnotes alongside variable components (also in S3). When considering behaviours and time points separately, Appropriate use and cleaning were consistently above the *a priori* threshold of 50%. However when assessing throughout, findings are mixed. Appropriate use did not meet the threshold (48·0%) whilst cleaning did (56·0%) and only 26·0% of households appropriately used and cleaned the HPS throughout the trial. Variables associated with adherence outcomes across the two time points were assessed using a binary logistic GEE model (Table 4). Results display the 95% CI for the effect size and odds ratio. The only variable to significantly predict Appropriate use or cleaning was 'Mother collects water alone', where an inverse relationship showed a reduced odds of 72·0% (0·28; 95% CI 0·12−0·66).

Table 3. Adherence: Appropriate playspace use and cleaning across study time points.

**Adherence: Appropriate HPS use and cleaning (N=50)**									
	Two weeks			Four weeks			Both time points[γ]		
	n	%	95% CI	n	%	95% CI	n	%	95% CI
Appropriate use[*]	35	70·0	55·5–82·1	32	64·0	49·2–77·1	24	48·0	33·7–62·6
Appropriate cleaning[**]	36	72·0	57·5–83·8	35	70·0	55·4–82·1	28	56·0	41·3–70·0
Appropriate use and cleaning[α]	26	52·0	37·4–66·3	24	48·0	33·7–62·6	13	26·0	14·6–40·[illegible]

HPS, household playspace; CI, confidence interval.
[*]Created from the variables: Playspace is assembled correctly (observed), yes; Any changes/modifications to playspace (observed), no, or yes, modifications are safe; Others share playspace (reported), no; Animals in playspace (observed and reported), no; Caregiver leaves infant in playspace when leaving house (reported), no or yes IF; infant is watched by other adult (father, grandparent or child ≥18).
[**]Created from the variables: Frequency of cleaning the playspace (reported), every day, every other day; Cleaning materials used (reported), water and soap; Mattress visibly dirty (observed), no; Urine or faeces on mattress (human or animal; reported), no.
[α]The sum of households who achieved 'Yes' for all criteria for both use and cleaning.
[β]The sum of households who achieved 'No' for all criteria for both use and cleaning.
[γ]The sum of households who achieved 'Yes' for all criteria across indicators at both two and four weeks.

Table 4. Adherence: A Generalised Estimation Equation estimating the effect of parameters on the trial outcome of adherence 'Appropriate use and cleaning' across study time points.

**Adherence: Appropriate use and cleaning Generalised Estimating Equation (N=50)**							
**Variable**			95% Wald Confidence Interval			95% Wald Confidence Interval for Exp(B)	
	B	Std. Error	Lower	Upper	Odds Ratio	Lower	Upper
(Intercept)	-0·07	1·06	-2·14	2·01	0·94	0·12	7·49
Infant sex = Male	0·42	0·50	-0·57	1·40	1·52	0·57	4·06
Maternal age = ≤25	-0·65	0·70	-2·02	0·71	0·52	0·13	2·04
Maternal education = Illiterate	-0·56	0·61	-1·75	0·63	0·57	0·17	1·88
Number in household = 1–3	-0·0	0·78	-2·52	0·52	0·37	0·08	1·68
Number of children = 1-2	0·45	0·59	-0·71	1·60	1·56	0·49	4·96
Household owns soap = 1	0·63	0·53	-0·41	1·67	1·87	0·66	5·31
Water is safely stored = Yes	-0·11	0·54	-1·17	0·95	0·90	0·31	2·59
Animals inside day = Yes	-0·22	0·53	-1·26	0·81	0·89	0·28	2·25
Animals inside night = Yes	-0·38	0·69	-1·73	0·97	0·68	0·18	2·63
Water available = Yes	-0·55	0·75	-2·03	0·93	0·58	0·13	2·54
Mother collects water alone=Yes	-1·28	0·44	-2·15	-0·42	0·28	0·12	0·66
Infant age (scale)	0·12	0·10	-0·07	0·31	1·13	0·93	1·37

Acceptability

Infant in playspace upon arrival, change in playspace use

The first measure noted if the infant was in the HPS during a random visit (Table 2). This increased from 32·0% (95% CI 19·5−46·7, n=16) at two weeks to 50% (95% CI 35·5−64·5, n=25) at four weeks, meeting *a priori* criteria of 50% at this point: however throughout the trial did not reach the threshold (41·0%, 95% CI 31·3−51·3; n=41). Second, change in incidence (as a proportion) of HPS during daily activities was assessed. Primary caregivers were asked open-ended questions to record their activities during the past 24 hours, and if they did or did not use the HPS. Results are shown in Table 5, with activities categorised. Broadly, there was no change in use throughout the trial during food preparation/eating but use increased during other activities inside the home (such as breastfeeding) and outside, such as preparing enset and farming. A full table describing activities and HPS use or non-use is in S4. Lastly analysing HPS use according to the time of day suggested use was consistently highest in the mornings, although evening use increased at the trial end (S5).

Table 5. Acceptability: Reported playspace use in the past 24 hours during different daily activities, at two and four weeks, and the change across time points.

Acceptability: Reported HPS use during specified household activities by 24-hour recall and change in proportion of use across study time points (N=50)

Reported daily activity	Total reported activity[*]	Reported HPS use	Proportion of use[**]	Total reported activity*	Reported HPS use	Proportion of use [**]	**Change in use**	**Change in proportion of use**[α]
	Two weeks			Four weeks			Across time points	
	n	n	%	n	n	%	n	%
**Prepared / ate a meal**	**150**	**139**	**92·7**	**172**	**139**	**80·8**	**0**	**-11·9**
Prepared breakfast	45	40	88·9	47	42	89·4	2	+0·5
Prepared lunch/snacks	56	54	96·4	51	48	94·1	-6	-2·3
Prepared dinner	49	45	91·8	49	43	87·8	-2	-4·1
Ate a meal	0	0	0·0	25	6	0·0	6	0·0
**Prepared coffee**	**75**	**69**	**92·0**	**95**	**85**	**89·5**	**16**	**-2·5**
**Duties within the home**	**57**	**46**	**80·7**	**73**	**62**	**84·9**	**16**	**+4·2**
Cleaned the house	47	40	85·1	55	47	85·5	7	+0·3
Washed clothes	10	6	60·0	18	15	83·3	9	+23·3
**Duties outside of the home**	**43**	**35**	**81·4**	**49**	**43**	**87·8**	**8**	**+6·4**
Fetched water	41	40	97·6	48	44	91·7	4	-5·9
Prepared enset	30	25	83·3	20	19	95·0	-6	+11·7
Chopped wood	7	6	85·7	8	6	75·0	0	-10·7
Farmed / maintained shop	6	4	66·7	21	18	85·7	14	+19·0
**Visits outside home**	**23**	**17**	**73·9**	**32**	**15**	**46·9**	**-2**	**-27·0**
Went to church / meeting	4	1	25·0	4	0	0·0	-1	-25·0
Went to market	15	14	93·3	16	12	75·0	-2	-18·3
Visited neighbours/ other	4	2	50·0	12	3	25·0	1	-25·0
**Breastfed / fed baby**	**26**	**15**	**57·7**	**30**	**20**	**66·7**	**5**	**+9·0**
**Slept / rested**	**23**	**5**	**21·7**	**18**	**3**	**16·7**	**-2**	**-5·1**

HPS, household playspace.
[*]Number represents reported incidence of that activity within the past 24 hours. Households (N=50) were asked an open-ended question about their daily activities during the past 24 hours. Not every activity was reported by every respondent.
[**]Calculated as the proportion of households who reported using the HPS during that daily activity.
[α]Calculated as the difference between the proportions of HPS use at two and four weeks.

Modified Barrier Analysis

Acceptability was further assessed through a semi-structured questionnaire as a modified Barrier Analysis. This assessed 12 categories of behavioural determinants, exploring all factors which would act as barriers or enablers during a definitive trial (S1). Full results are in S6. The first seven determinants quantitatively assess beliefs and behaviours relating to infant health and HPS use. The further six determinants explored attitudes and beliefs through open-ended questions. Many cited advantages, both related and unrelated to infant health, indicated good acceptability of the HPS. Caregivers frequently stated the HPS helped prevent ingestion of dirt and faeces (80·0%, n=40), 76·0% (n=38). Further, many suggested the HPS prevented injury from several causes, including from fire, drowning and animals. Over half of caregivers (mothers) asserted that the HPS eased their workload (56·0%, n=28), reduced time pressures (46·0%, n=23) and allowed them to carry out their duties without distraction. Mothers reported relief that the HPS alleviated fears and worries over their infant's safety (52·0%, n=26), and almost half believed their infant would physically grow better (42·0%, n=21). Approval within the community was high among neighbours (96·0%, n=48) husbands (40·0%, n=20), and both close (66·0%, n=33) and wider family (36·0%, n=18). Conversely, some caregivers mentioned that neighbours (8·0%, n=4) or friends (12·0%, n=6) were envious as the common reason for disapproval *('My friend who does not have one wants one too')*, or that money would have been preferable *('My colleague says better to give the child clothes or money for me')*.

Barriers to use included the cost of cleaning materials (22·0%, n=11) – echoed in the *Access* determinant where caregivers frequently noted the expense of soap (56·0%, n=28) and cleaning materials, e.g. brushes (24·0%, n=12). Importantly, having no older children to watch the infant was a barrier (32·0%, n=16) and relates to the burden of workload on women. A lack of toys was also a barrier (32·0%, n=16). Whilst the design appeared largely acceptable, some difficulties included fitting the rope connecting walls (38·0%, n=19; see S3).

Secondary outcomes: Infant health outcomes

Table 6 shows changes in reported seven-day diarrhoeal prevalence and presumptive *Campylobacter* spp. across groups and time periods. Considering change in point prevalence, seven-day diarrhoea declined more markedly within the intervention group from 19 cases (38·0%) at baseline to 5 cases (10.0%) at four weeks, versus 22 cases (44·0%) to 16 (32·0%) amongst controls. Considering change in prevalence from baseline, the intervention group showed a reduced odds of reported diarrhoea versus controls (OR 0·57, 95% CI 0·40−0·83). Baseline prevalence of presumptive C*ampylobacter* was high, mirroring a similar prevalence at this site and others.[26,29] However from baseline, point prevalence showed no significant difference between groups or time points. Similarly the intervention group had no reduced odds of a *Campylobacter*-positive stool versus controls from baseline. Colony counts from positive samples can be viewed in S7.

Table 6. Secondary health outcomes: Point prevalence across study time points and change in prevalence from baseline for seven-day diarrhoea and Campylobacter, intervention and control.

**Secondary outcomes: Change in infant health outcomes**												
**Reported seven-day diarrhoea point prevalence across study time points**												
	Baseline				Two weeks				Four weeks			
	Intervention (n=50)		Control (n=50)		Intervention (n=50)		Control (n=50)		Intervention (n=50)		Control (n=50)	
	n	%	n	%	n	%	n	%	n	%	n	%
No diarrhoea	31	62·0	28	56·0	44	88·0	35	70·0	45	90·0	34	68·0
Diarrhoea	19	38·0	22	44·0	6	12·0	15	30·0	5	10·0	16	32·0
**Presumptive *Campylobacter* point prevalence across study time points**												
No infection	23	46·0	24	48·0	33	66·0	32	64·0	36	72·0	36	72·0
Infection	27	54·0	26	52·0	17	34·0	18	36·0	14	28·0	14	28·0

**Change in reported seven-day diarrhoeal prevalence after baseline[*]**				
	Intervention (n=50)		Control (n=50)	
	n	%	n	%
No diarrhoea[**]	39	78·0	28	56·0
Diarrhoea	11	22·0	22	44·0
**Change in presumptive *Campylobacter* prevalence after baseline[β]**				
No infection[α]	30	60·0	28	56·0
Any infection	20	40·0	22	44·0

[*]OR for intervention group 0·49 (95% CI 0·33–0·75)
[**]No diarrhoea: No reported diarrhoea at two or four weeks, OR no reported diarrhoea from baseline; Diarrhoea: Reported diarrhoea at two or four weeks, OR reported diarrhoea from baseline.
[β]Insignificant.
[α]Negative: No suspected *Campylobacter* at two or four weeks, OR always negative; Positive: Suspected *Campylobacter* prevalence at two or four weeks, OR always positive.

Harms

No adverse events were observed from HPS use in the intervention group. No household reported any safety concerns associated with use, aside from one household who mentioned the plastic mattress became hot under the sun. HPS use did not increase the risk of any adverse infant health outcome, where the direction of effect does not show an increased risk for the intervention group.

Discussion

The CAMPI trial is the first randomised, controlled feasibility trial of a HPS in rural, subsistence agriculture households in Ethiopia. Though trial outcomes did not fully reach *a priori* criteria, a definitive RCT for efficacy is feasible. Results echo two similar studies. In the SHINE trial in Zimbabwe, an imported plastic HPS and locally-sourced plastic playmat were included in a WASH intervention to improve growth and anaemia. Whilst fidelity of delivery was high,[10] the WASH intervention did not prevent infection.[30] However, the analysis did not estimate a magnitude of effect from the HPS specifically. In Zambia, a community-built HPS was assessed alongside a plastic model for acceptability and feasibility.[31] Reported use was similar between the two types (ranging from 10 minutes to three hours), family and community reactions suggested acceptability was high and caregiver reports suggested the community built space prevented infant ingestion of soil and animal faeces. Thus growing evidence supports wide acceptability and feasibility across different contexts and further rigorous assessment of efficacy is merited.

Addressing barriers to appropriate use and cleaning of the HPS would improve these outcomes. Data here described a broadly consistent pattern over the four weeks, albeit with a small decline (Tables 2 and 3). The modified Barrier Analysis offered reasons for diminishing use and drops in compliance, including the expense of soap and other cleaning materials. Providing these alongside the HPS would be a key consideration for any future RCT to ensure good hygiene. Similarly, contextual WASH factors, such as water quality, availability, and unsafe storage (76·0%, n=38 in the intervention group; Table 1) must be considered which may result in increased bacterial transmission. Similarly, the team

decided not to provide toys during the trial given the potential to become vectors for indirect faecal-oral transmission.[18,32] However, this was a frequently cited barrier for mothers whose infants became bored and cried: thus providing toys or including stimulating features to the HPS is an important consideration. Alternatively caregivers may be counselled on providing (non-porous), non-hazardous toys and on regular proper cleaning. Further, during early, critical growth periods there are other important considerations including psychosocial and neurodevelopment. Opportunities for linguistic, socioemotional, and cognitive development are critical and a future RCT should consider if a HPS reduces these opportunities through interruptions to normal play, exploration, and caregiver-infant interaction – all strongly related to contextual norms and traditions.

Through random spot checks of HPS use and change in time-use, the trial showed mixed acceptability, partly meeting *a priori* criteria (Tables 2 and 5). Reported daily use increased for certain activities, suggesting an increasing ease with incorporating the HPS into daily life. However increased use during certain activities (fetching water, farming) may indicate a complacency with infant safety inside the HPS and present a risk. These increases may account for the reduction in 'Appropriate use' at week four which included if the caregiver left the infant alone whilst outside. A key finding from the modified Barrier Analysis were the secondary effects of easing work burden, time restraints, and worries about infant health and safety for many mothers. Thus in the short term, a HPS may hold many benefits including potentially improving women's empowerment through time availability and choice, reducing anxiety, and even freeing up time to spend with her infant. However any negative long-term impacts will need to balance these. This includes a lack of infant supervision, and the risk of reinforcing women's roles as sole caregivers alongside a continuing responsibility for other domestic duties. This is reinforced by the GEE model (Table 4) where when the mother bore the duty of collecting water alone, the HPS was less likely to be used or cleaned properly. In many low-income countries, women's' 'triple work burden' in the productive, reproductive and social domains impedes their well-being and may reduce engagement in childcare[33] – a pattern often inherited by older female siblings. This highlights a trade-off in encouraging more active parenting alongside existing home duties, and any intervention must ensure it does not further encumber women.

The CAMPI trial was not powered to detect any differences in health outcomes between groups and results should be interpreted accordingly. However secondary infant health outcomes indicated the potential efficacy of a HPS and appropriateness of these outcomes for a future RCT. Diarrhoeal prevalence from baseline reduced among the intervention group whilst presumptive *Campylobacter* did not (Table 6). Beyond the lack of adequate power, substantial methodological limitations may profoundly affect validity: these include the reliability of caregiver-reported diarrhoea and a desirability bias within intervention households. No further GEE analysis was performed to explore associated variables. However, aside from a potential lack of effect of the HPS on *Campylobacter* prevalence, other pathways not interrupted by the HPS likely contributed to pathogen transmission. This includes incorrectly (re-)heated foods[34]; data on this indicated unsafe practices were common (S8) where across households only 28 safely prepared all meals at both time points (data not shown). All infants were given liquids other than breastmilk, including water, possibly contaminated through unsafe storage or other pathways. *Campylobacter* from domestic free-range poultry appears to present an infection risk to infants[5,26] and here poultry frequently shared living and sleeping areas (Table 1). However, questions remain on what, how and where infants contract *Campylobacter*, the role of domestic animals in transmission and survival time in the environment.[34] The methodology used to isolate *Campylobacter* spp. also holds limitations[26] and a definitive RCT should consider other, more sensitive techniques such as the use of ELISA or quantitative PCR.

Progression to a definitive RCT

To improve playspace adherence and acceptance, a future definitive RCT should focus on directly addressing the barriers whilst promoting the enabling factors as identified in this feasibility trial. Whilst further behavioural 'modules' and developing caregiver knowledge might have improved outcomes, it is not always practical. During the sensitisation day the HPS was introduced in a 'scalable' manner to reduce work burden among households and HEWs who are already overworked. Rather, to achieve behavioural change it is pragmatic to directly address barriers and promote enabling factors. Knowledge alone is unlikely to prevent infant faecal-oral transmission without a

material element which breaks contact, and an enabling technology may drive changes in behaviour but still requires addressing factors which support or obstruct change. Factors included in the composite variable Appropriate use responsible for a decline include another child sharing the HPS (S3). Given the potential to introduce contamination, this might be addressed by a visiting HEW as a risk factor. Similarly, Appropriate cleaning declined from every day/every other day to twice a week. The direction of effect and significance in the GEE model (Table 4) is an important consideration to improving this: cleaning behaviours will not change without access to soap. To improve time-use, toys (non-porous) might be provided with counselling from HEWs on regular cleaning. Factors not modifiable to counselling are important prognostic factors and might be included as strata in group randomisation in a full RCT.

Several contextual factors undoubtedly influenced this trial's operational success, including ease of recruitment and full retention. The study kebeles, within PIN outreach, may have resulted in higher acquiescence during recruitment and consent. High retention likely results from this plus a high number of data collectors for the sample. However, it is important to note that daily data collection was intense and required serious team dedication. A larger trial would likely experience higher drop out without equivalent input: a 95% CI estimate would be between 96−100% in a power calculation and 95−100% if repeated maintaining the same effort and ratio of study personnel. Over a longer time period, this is likely unsustainable. Future sample size calculations must consider these number requirements for study personnel. Furthermore, as recipients of previous WASH interventions, the intervention group likely adopted the new intervention modality earlier than might be seen in other contexts, holding implications for external validity. Good uptake may also be seen in other contexts where NGOs have a known presence and have provided multiple interventions for many years, but this does limit the generalisability of findings to other contexts. Lastly it is important to note the extensive HPS design process and the underlying formative work. Good contextual understanding is critical for intervention success, which must be culturally acceptable, locally integrated and must consider contextual baseline demographic and WASH characteristics and health status which vary significantly.

Conclusion

The CAMPI trial evaluated feasibility of a BabyWASH HPS and recommendations to progress to a future RCT in a rural, subsistence agriculture setting in Ethiopia. Not all *a priori* criteria were met. Overall the HPS showed mixed engagement and adherence, good acceptability and many reported secondary benefits. A HPS remains a viable option to help reduce direct faecal-oral transmission and infant infection in these settings. A larger trial with longer follow-up is feasible to implement and should assess infant health outcomes as primary endpoints. Addressing identified barriers and promoting enabling factors would likely improve adherence and use.

Acknowledgements

This study would not have been possible without the tireless efforts of the data collection teams, including Mesfine Melese, Metsinanat Eyoel, Wonsha Bulbula, Dawit Daniso, Deginet Aklilu and Biruk Solomon. Particular special thanks go to Etsegenet Yisak Debela and Abezash Asefa Wotasa as laboratory assistants at Hawassa University College of Medicine and Health Sciences who dedicated their energy to the study. We are grateful to Afework Abraham, Frezer Girma and Endale Eyob at People In Need who kept the teams running daily. Thanks also go to the Health Extension Workers who supported the data collection teams. Finally, we thank all of the study participants who gave their valuable time and input throughout the trial duration.

Author contributions

SB was mainly responsible for the trial design and oversight and principally wrote the manuscript. PH, AP and CG contributed towards study design and assisted in assessing the paper quality, writing and review. SN assisted with statistical analysis and in assessing paper quality and review. FW and MJ provided input with the study design, planning and logistics and management of the field team during the trial. MM, SHA and HB assisted in reviewing the paper and were valuable partners at Hawassa University College of Medicine and Health Sciences, overseeing laboratory practice.

Funding

SB is jointly funded as a research student by both Cranfield University and People In Need, who received funding from the Czech Development Agency for the project. No other external funds supported this work.

Declaration of interest

All authors have no conflict of interest to declare.

Data access and sharing

All of the individual participant data collected during the trial, after de-identification, will be available immediately following publication and with no end date, for any analysis purpose. Data are available indefinitely, accessible online at the Cranfield University data repository. The study protocol and informed consent form are also available upon request.

Supporting information

CONSORT checklist

CONSORT participant flow diagram

References

1. Millward D. Nutrition, infection and stunting: The roles of deficiencies of individual nutrients and foods, and of inflammation, as determinants of reduced linear growth of children. *Nutr Res Rev*. 2017;30(1):50–72.
2. Nataro JP, Guerrant RL. Chronic consequences on human health induced by microbial pathogens: Growth faltering among children in developing countries. *Vaccine*. 2017;35:6807–6812. doi:10.1016/j.vaccine.2017.05.035
3. Watanabe K, Petri W. Environmental Enteropathy: Elusive but Significant Subclinical Abnormalities in Developing Countries. *EBio Med*. 2016;10:25–32.

4. MAL-ED Network Investigators. Relationship between growth and illness, enteropathogens and dietary intakes in the first 2 years of life: Findings from the MAL-ED birth cohort study. *BMJ Glob Heal*. 2017;2(4):e000370. doi:10.1136/bmjgh-2017-000370
5. Lee G, Pan W, Penataro Yori P, et al. Symptomatic and Asymptomatic Campylobacter Infections Associated with Reduced Growth in Peruvian Children. *PLoS Neglected Trop Dis*. 2013;7(1):e2036. doi:10.1371/journal.pntd.0002036
6. Unger SA, Drammeh S, Hasan J, et al. Impact of fortified versus unfortified lipid-based supplements on morbidity and nutritional status: A randomised double-blind placebo-controlled trial in ill Gambian children. *PLoS Med*. 2017;14(8):e1002377. doi:10.1371/journal.pmed.1002377
7. Fink G, Günther I, Hill K. The effect of water and sanitation on child health: Evidence from the demographic and health surveys 1986-2007. *Int J Epidemiol*. 2011;40(5):1196–1204.
8. Luby SP, Rahman M, Arnold BF, et al. Effects of water quality, sanitation, handwashing, and nutritional interventions on diarrhoea and child growth in rural Bangladesh: a cluster randomised controlled trial. *Lancet Glob Heal*. 2018;6(3):e302-e315. doi:10.1016/S2214-109X(17)30490-4
9. Null C, Stewart CP, Pickering AJ, et al. Effects of water quality, sanitation, handwashing, and nutritional interventions on diarrhoea and child growth in rural Kenya: A cluster-randomised controlled trial. *Lancet Glob Heal*. 2018;6(3):e316–e329. doi:https://doi.org/10.1016/S2214-109X(18)30005-6
10. Humphrey, Mbuya MNN, Ntozini R, et al. Independent and combined effects of improved water, sanitation, and hygiene, and improved complementary feeding, on child stunting and anaemia in rural Zimbabwe: a cluster-randomised trial. *Lancet Glob Heal*. 2019;7(1):e132–e147. doi:10.1016/S2214-109X(18)30374-7
11. Clasen T, Boisson S, Routray P, et al. Effectiveness of a rural sanitation programme on diarrhoea, soil-transmitted helminth infection, and child malnutrition in Odisha, India: A cluster-randomised trial. *Lancet Glob Heal*. 2014;2(11):e645-e653. doi:10.1016/S2214-109X(14)70307-9
12. Pickering A, Null C, Winch P, Mangwadu G, Arnold B, Prendergast A. The WASH Benefits and SHINE trials: interpretation of WASH intervention effects on linear growth and diarrhoea. 2019;7(8):E1139-E1146. doi:10.1016/S2214-109X(19)30268-2
13. Penakalapati G, Swarthout J, Delahoy M et al. Exposure to Animal Feces and Human

Health: A Systematic Review and Proposed Research Priorities. *Envir Sci Technol*. 2017;51(20):11537–11552. doi:10.1021/acs.est.7b02811

14. Boehm AB, Wang D, Ercumen A, et al. Occurrence of Host-Associated Fecal Markers on Child Hands, Household Soil, and Drinking Water in Rural Bangladeshi Households. *Environ Sci Technol Lett*. 2016. doi:10.1021/acs.estlett.6b00382
15. Ercumen A, Pickering AJ, Kwong LH, et al. Animal Feces Contribute to Domestic Fecal Contamination: Evidence from E. coli Measured in Water, Hands, Food, Flies, and Soil in Bangladesh. *Environ Sci Technol*. 2017;51(15):8725-8734. doi:10.1021/acs.est.7b01710
16. George C, Oldja L, Biswas S et al. Fecal markers of environmental enteropathy are associated with animal exposure and caregiver hygiene in Bangladesh. *Am J Trop Med Hyg*. 2015;93(2):269-275. doi:10.4269/ajtmh.14-0694
17. Weisz AJ, Manary MJ, Stephenson K, et al. Abnormal gut integrity is associated with reduced linear growth in rural malawian children. *J Pediatr Gastroenterol Nutr*. 2012;55(6):747-750. doi:10.1097/MPG.0b013e3182650a4d
18. Ngure F, Humphrey J, Mbuya M et al. Formative research on hygiene behaviors and geophagy among infants and young children and implications of exposure to fecal bacteria. *Am J Trop Med Hyg*. 2013;89(4):709–716. doi:10.4269/ajtmh.12-0568
19. WHO/UNICEF. *Position Paper: Implications of Recent WASH and Nutrition Studies for WASH Policy and Practice*.; 2019. https://www.who.int/water_sanitation_health/news-events/reflections-series/en/.
20. Morita T, Perin J, Oldja L et al. Mouthing of Soil Contaminated Objects is Associated with Environmental Enteropathy in Young Children. *Trop Med Int Heal*. 2017;22(6):670–678. doi:10.1111/tmi.12869
21. Reid B, Orgle J, Roy K, Pongolani C, Chileshe M, Stoltzfus R. Characterizing potential risks of fecal–oral microbial transmission for infants and young children in Rural Zambia. *Am J Trop Med Hyg*. 2018. doi:10.4269/ajtmh.17-0124
22. Pickering A, Julian T, Marks S et al. Fecal contamination and diarrheal pathogens on surfaces and in soils among Tanzanian households with and without improved sanitation. *Envir Sci Technol*. 2012;46(11):5736–5743. doi:10.1021/es300022c
23. Budge S, Hutchings P, Parker A, et al. Multisectoral participation in the development of a BabyWASH playspace for rural Ethiopian households. *In prepration*. 2020.
24. Budge S, Parker A, Hutchings P, Garbutt C. Environmental enteric dysfunction and child stunting. *Nutr Rev*. 2019;77(4):240–253. doi:10.1093/nutrit/nuy068

25. Budge S, Hutchings P, Parker A, et al. Do domestic animals contribute to bacterial contamination of infant transmission pathways? Formative evidence from rural Ethiopia. *J Water Health*. 2019;17(5). doi:10.2166/wh.2019.224

26. Budge S, Barnett M, Hutchings P, et al. Domestic poultry ownership is associated with infant Campylobacter spp. infection and malnutrition: formative evidence from Ethiopia. *PLoS One*. 2019;15(5):e0232541. doi:https://doi.org/10.1371/journal.pone.0232541

27. de Onis M, Onyango AW, Van den Broeck J, Chumlea WC, Martorell R. Measurement and standardization protocols for anthropometry used in the construction of a new international growth reference. *Food Nutr Bull*. 2004;25(1):S27-S36.

28. WHO Multicentre Growth Reference Study Group. *WHO Child Growth Standards. Length/Height-for-Age, Weight-for-Age, Weight-for-Length, Weight-for-Height and Body Mass Index-for-Age: Methods and Development. Department of Nutrition Health and Development.* Geneva: World Health Organization; 2006.

29. Sanchez J, Alam A, Stride C, et al. Campylobacter infection and household factors are associated with childhood growth in urban Bangladesh: An analysis of the MAL-ED study. *PLoS Negl Trop Dis*. 2020;15(5):e0008328.

30. Rogawski McQuade ET, Platts-Mills JA, Gratz J, et al. Impact of Water Quality, Sanitation, Handwashing, and Nutritional Interventions on Enteric Infections in Rural Zimbabwe: The Sanitation Hygiene Infant Nutrition Efficacy (SHINE) Trial. *J Infect Dis*. 2019;221(8):1379-1386. doi:10.1093/infdis/jiz179

31. Reid, Rie S, Jennifer O, et al. A community-designed play-yard intervention to prevent microbial ingestion: A Baby WASH pilot study in rural Zambia. *Am J Trop Med Hyg*. 2017;99(2):513–525.

32. Vujcic J, Ram P, Hussain F et al. Toys and toilets: Cross-sectional study using children's toys to evaluate environmental faecal contamination in rural Bangladeshi households with different sanitation facilities and practices. *Trop Med Int Heal*. 2014;19(5):528–536.

33. Grassi, Flavia; Landberg, Josefine; Huyer S. *Running out of Time: The Reduction of Women's Work Burden in Agricultural Production*. Rome, Italy: Food and Agriculture Organization of the United Nations; 2015.

34. Asuming-Bediako N, Parry-Hanson Kunadu A, Abraham S, Habib I. Campylobacter at the Human–Food Interface: The African Perspective. *Pathogens*. 2019;8(2):87. doi:10.3390/pathogens8020087

609

Supplementary information

S1. Modified Barrier Analysis methodology

Background to a Barrier Analysis

A Barrier Analysis (BA) is the main type of formative research recommended in the Designing for Behavioural Change framework. It is described as the 'field research step' with a primary aim to identify what is preventing the target groups from practicing the targeted behaviours (the 'barriers') and what might encourage adoption (the 'motivators').[1] The full official training syllabus, published by Kittle BL, provides a practical guide.[2] Briefly, the standard BA method asks the target group a series of questions aiming to identify barriers and motivators, using a Doer/Non-Doer methodology. This consists of interviewing the group who already do the behaviour and the group who have not yet adopted the behaviour. The motivators and barriers identified in the survey are categorized under 1 of 12 possible determinants. These determinants represent the perceptions, feelings, and beliefs of the target group and determine why they do or do not adopt a specific behaviour and are as follows: Perceived self-efficacy; Perceived social norms; Perceived positive consequences; Perceived negative consequences; Access; Cues for action/reminders; Perceived susceptibility/risk; Perceived severity; Perceived action efficacy; Perceived divine will; Policy and Culture. Analysing the difference between the Doers' and Non-Doers' responses indicates which barriers or enabling factors are the most important in determining that behaviour. Insights into these perceptions are used to further develop the content and strategies of the behavioural change activity. Such links are called 'Bridges to Activities'.

Barrier Analysis method in the CAMPI feasibility trial

Given that there were no 'non-doers' in the CAMPI feasibility trial, intervention households were not analysed according to the standard Doer/Non-doer methodology as described above. Rather, the methodology was modified slightly to explore behaviours among all participants, without categorising them as Doers/Non-doers. The survey used in the modified BA is shown below. Intervention households were interviewed by the data collection teams, trained in the BA method, alongside a Health Extension Worker at week four of the feasibility trial. Following data collection, responses were entered into Excel. Questions 2−9 were entered into categories according to determinants and then categorised into themes which arose from the data. Those themes were summed and described in the results. Quantitative responses (questions 1, 10−16 in the table below) were summed in each answer category and also subsequently described in the result. As such, the determinants allowed for the description of barriers/enabling factors among all households which would improve adherence to a greater degree – given that all households used the playspace during the trial.

1. Schmied, P 2017. Behavioural Change Toolkit for International Development Practitioners. People In Need: Prague, Czech Republic.

2. Kittle, B. 2017. A Practical Guide to Conducting a Barrier Analysis (2nd ed.). Helen Keller International: New York, NY.

1	Do you think that you use the play space for your child whenever you can?	Yes<br>No
2	What are the **advantages** of using the play space?	
3	What are the **disadvantages** of using the play space?	
4	What makes it **easy** for you to use the play space?	
5	What makes it **difficult** for you to use the play space?	
6	What makes it **easy** for you to keep the play space clean?	
7	What makes it **difficult** for you to keep the play space clean?	
8	Who are the people who **approve** of you using the play space for your child?	
9	Who are the people who **disapprove** of you using the play space for your child?	
10	How difficult is it to remember to use the play space for your child every time you could use it?	Very difficult<br>A bit difficult<br>Not difficult<br>Don't know
11	How likely do you think it is your child will get diarrhoeal disease within the next month?	Very likely<br>Quite likely<br>Not likely<br>Don't know
12	How serious would it be if your child had a diarrhoeal disease?	Very serious<br>Quite serious<br>Not serious<br>Don't know
13	How likely do you think it is your child will get diarrhoeal disease if you used the play space whenever you could?	Very likely<br>Quite likely<br>Not likely<br>Don't know
14	Do you think God approves of you using the play space?	Yes<br>No<br>Don't know<br>Specify:
15	Are there any community rules which prevent you from using the play space?<br>*If yes,* what are they?	Yes<br>No<br>Don't know<br>Specify:
16	Are there any cultural rules that you know of against using the play space?<br>*If yes,* what are they?	Yes<br>No<br>Don't know<br>Specify:
17	Ask the caregiver:<br>*Do you have any other comments about the play space, positive **OR** negative?*	

S2. The prototype design of the BabyWASH household playspace used in the CAMPI trial.

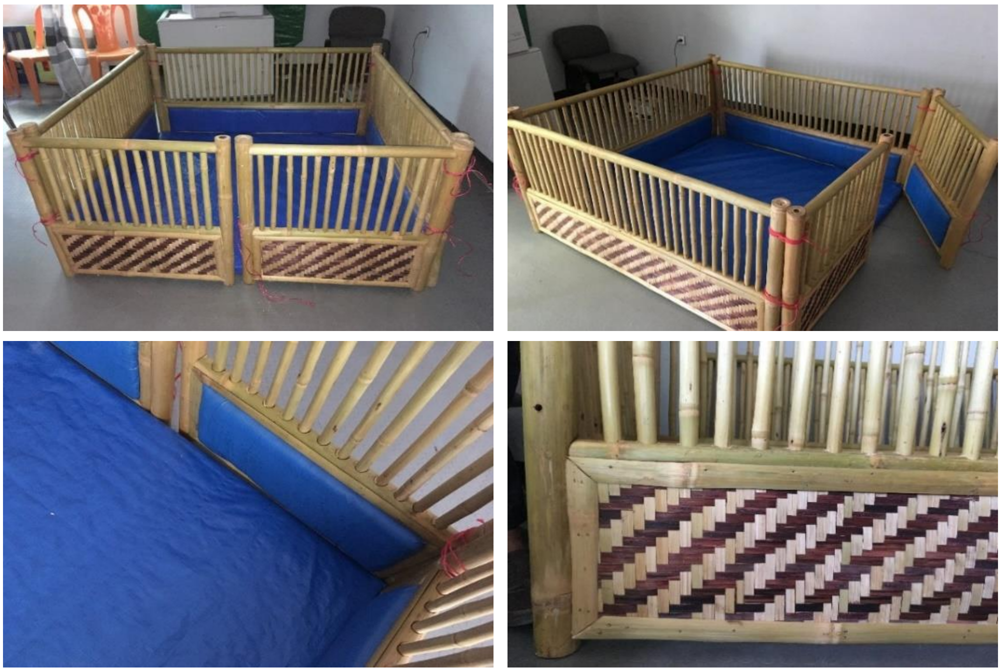

S3. Playspace use behaviours and infant hygiene and playspace cleaning practices included as part of composite variables 'Appropriate use' and 'Appropriate cleaning'.

**HPS use behaviours**						
	Two weeks		Four weeks		Both time points	
	n (50)	%	n (50)	%	N (100)*	%**
Who watches the infant: Another child	42	84·0	43	86·0	85	85·0
Mother	27	54·0	28	46·0	55	55·0
Husband	18	36·0	24	48·0	42	42·0
A grandparent	0	0·0	1	2·0	1	1·0
Infant in HPS when leave house	41	82·0	26	52·0	67	67·0
Other child shares the HPS	14	28·0	18	36·0	32	32·0
Who shares the HPS: Mother to feed	6	12·0	10	20·0	16	16·0
Sister or brother	4	8·0	4	8·0	8	8·0
Another child	2	4·0	6	12·0	8	8·0
Twin	1	2·0	1	2·0	2	2·0
Infant given toys or items to play	43	86·0	46	92·0	89	89·0
Items given: Plastic cup	32	64·0	33	66·0	65	65·0
Plastic water bottle	27	54·0	27	54·0	54	54·0
Jerry can cover	8	14·0	6	12·0	14	14·0
Empty plastic container	7	13·0	5	10·0	12	12·0
Mobile phone	6	12·0	6	12·0	12	12·0
Small ball	5	10·0	5	10·0	10	10·0
Store-bought plastic toys	2	4·0	6	12·0	8	8·0
Book/paper	2	4·0	2	4·0	4	4·0
Reasons to remove infant: Infant hungry	49	98·0	49	98·0	98	98·0
Infant is crying (bored)	44	88·0	46	92·0	90	90·0
Infant has defecated/urinated	39	78·0	37	74·0	76	76·0
To clean the playspace	30	60·0	26	52·0	56	56·0
To wash/change infant	25	50·0	30	60·0	55	55·0
To breastfeed/feed	11	22·0	15	30·0	26	26·0
Infant is sleeping	4	8·0	1	2·0	5	5·0
To go out	1	2·0	2	4·0	3	3·0
**Infant hygiene and HPS cleaning**						
**Observational data**						
Infant visibly dirty upon arrival	20	40·0	19	38·0	39	39·0
Infant has dirty hands and nails	28	56·0	24	48·0	52	52·0
Visible dirt on mattress	6	12·0	3	6·0	9	9·0
Urine or faeces on mattress	1	2·0	1	2·0	2	2·0
Animals inside HPS (observed)	1	2·0	0	0·0	1	1·0
**Caregiver-reported data**						
How often clean HPS: Every day	30	60·0	16	32·0	46	46·0
Twice a week	9	18·0	17	34·0	26	26·0
Every other day	6	12·0	6	12·0	12	12·0
Only when infant defecates/urinates	3	6·0	11	22·0	14	14·0
Only when it is dirty	2	4·0	0	0·0	2	2·0
Cleaning materials used: Water only	5	10·0	2	4·0	7	7·0
Water and soap	45	90·0	48	96·0	93	93·0
Animals seen inside HPS: Yes	4	8·0	0	100·0	4	4·0
Which animals? Cat	2	50·0*	0	0·0	2	2·0
Poultry	2	50·0*	0	0·0	2	2·0

HPS, household playspace.
*Calculated as a cumulative total of both time points.
**Percent is of the cumulative total.

S4. Reported daily activities and reported use or non-use of the playspace during the past 24 hours, across daily periods and study time points.

	Morning								Afternoon								Evening							
	Two weeks				Four weeks				Two weeks				Four weeks				Two weeks				Four weeks			
	Used HPS		Did not use HPS		Used HPS		Did not use HPS		Used HPS		Did not use HPS		Used HPS		Did not use HPS		Used HPS		Did not use HPS		Used HPS		Did not use HPS	
	n*	%**	n	%	n	%	n	%	n	%	n	%	n	%	n	%	n	%	n	%	n	%	n	%
Prepared breakfast	40	89·0	5	11·0	42	89·0	5	11·0	0	0·0	0	0·0	0	0·0	0	0·0	0	0·0	0	0·0	0	0·0	0	0·0
Prepared coffee	32	94·0	2	6·0	37	95·0	2	5·0	5	83·0	1	17·0	14	88·0	2	12·0	32	91·0	3	9·0	34	85·0	6	15·0
Cleaned the house	37	86·0	6	14·0	35	83·0	7	17·0	3	75·0	1	25·0	9	100·0	0	0·0	0	0·0	0	0·0	3	75·0	1	25·0
Fetched water	22	96·0	1	4·0	17	85·0	3	15·0	18	100·0	0	0·0	26	96·0	1	4·0	0	0·0	0	0·0	1	100·0	0	0·0
Prepared lunch/snacks	0	0·0	0	0·0	0	0·0	0	0·0	48	96·0	2	4·0	44	98·0	1	2·0	6	100·0	0	0·0	4	67·0	2	33·0
Prepared enset	9	82·0	2	18·0	7	100·0	0	0·0	16	84·0	3	16·0	12	92·0	1	7·0	0	0·0	0	0·0	0	0·0	0	0·0
Washed clothes	2	50·0	2	50·0	1	33·0	2	67·0	4	67·0	2	33·0	12	82·0	1	7·0	0	0·0	0	0·0	2	100·0	0	0·0
Farmed / maintained shop	2	67·0	1	33·0	6	86·0	1	14·0	0	0·0	1	100·0	9	90·0	1	10·0	2	100·0	0	0·0	3	75·0	1	25·0
Prepared dinner	0	0·0	0	0·0	0	0·0	0	0·0	0	0·0	0	0·0	0	0·0	0	0·0	45	92·0	4	8·0	43	88·0	6	12·0
Went to church / meeting	1	100·0	0	0·0	0	0·0	4	100·0	0	0·0	3	100·0	0	0·0	0	0·0	0	0·0	0	0·0	0	0·0	0	0·0
Went to market	1	100·0	0	0·0	0	0·0	0	0·0	13	93·0	1	7·0	12	75·0	4	25·0	0	0·0	0	0·0	0	0·0	0	0·0
Breastfed / fed baby	7	88·0	1	12·0	7	70·0	3	30·0	5	63·0	3	37·0	4	57·0	3	43·0	3	30·0	7	70·0	9	69·0	4	31·0
Cleaned playspace	0	0·0	1	100·0	0	0·0	0	0·0	0	0·0	3	100·0	0	0·0	4	100·0	0	0·0	0	0·0	0	0·0	0	0·0
Washed infant	0	0	2	100·0	0	0·0	3	100·0	0	0·0	4	100·0	0	0·0	5	100·0	0	0·0	12	100·0	0	0·0	6	100·0
Chopped wood	0	0	0	0·0	0	0·0	0	0·0	6	86·0	1	14·0	6	75·0	2	25·0	0	0·0	0	0·0	0	0·0	0	0·0
Visited neighbours/ other	1	50	1	50·0	1	33·0	2	67·0	1	50·0	1	50·0	2	40·0	3	60·0	0	0·0	0	0·0	0	0·0	4	100·0
Ate a meal	0	0·0	0	0·0	0	0·0	0	0·0	0	0·0	0	0·0	0	0·0	2	100·0	0	0·0	0	0·0	6	26·0	17	74·0
Slept / rested	0	0·0	0	0·0	0	0·0	0	0·0	0	0·0	2	100·0	3	60·0	2	40·0	5	24·0	16	76·0	0	0·0	13	100·0

HPS, household playspace.
*Number represents reported incidence of that activity within the past 24 hours. Households (n=50) were asked an open-ended question on their daily activities during the past 24 hours. Not every activity was reported by every respondent.
**Percentages are calculated from the total number of households who reported that activity.

S5. Reported playspace use and non-use during daily activities in the past 24 hours across daily time periods: at two and four weeks.

	Morning		Afternoon		Evening	
	Two weeks	Four weeks	Two weeks	Four weeks	Two weeks	Four weeks
Reported use of HPS	154	153	119	153	93	105
Reported non-use of HPS	24	32	28	32	42	60

HPS, household playspace.
Figures are summed from reported daily activities table in S4.

S6. Modified Barrier Analysis results among the study intervention group.

BA determinant and question		Very…		Quite…		Not…		Don't know	
		n (50)	%	n (50)	%	n (50)	%	n (50)	%
Cues for action/ reminders	How **difficult** is it to remember to use the HPS every time you could?	0	0·0	0	0·0	50	100·0	0	0·0
Perceived susceptibility/ risk	How **likely** do you think it is your child will get diarrhoea within the next month?	0	0·0	9	18·0	24	48·0	17	34·0
Perceived severity	How **serious** would it be if your child had diarrhoea?	40	80·0	7	14·0	2	4·0	1	2·0
Perceived action efficacy	How **likely** is it your child will get diarrhoea if you used the HPS whenever you could?	0	0·0	9	18·0	38	76·0	3	6·0
		Yes		No		Don't know			
		n (50)	%	n (50)	%	n (50)	%		
Perceived divine will	Do you think God approves of you using the HPS?	48	96·0	1	2·0	0	0·0		
Policy	Are there any community rules which prevent you from using the HPS?	0	0·0	50	100·0	0	0·0		
Culture	Are there any cultural rules that you know of against using the HPS?	0	0·0	50	100·0	0	0·0		

HPS, household playspace.

BA determinant	BA question	Inductive theme	n (50)	%
Perceived positive consequences	What are the advantages of using the HPS?	Prevents ingestion of dirt/soil/dirty objects	40	80·0
		Prevents injury (falling, fire, drowning, dust/ash, road)	38	76·0
		Prevents injury from animals	29	58·0
		Decreases/Eases mother's workload	28	56·0
		Mother worries less for infant's health/safety	26	52·0
		Eases time pressure for mother/stress	23	46·0
		Improves infant's physical development	21	42·0
		Infant/Clothes stay clean	20	40·0
		Prevents ingestion of faeces	20	40·0
		Infant feels happy playing inside/comfortable	18	36·0
		Prevents diarrhoea/Other disease	14	28·0
		Protects from sunlight	4	8·0
		Promotes infant's independence	2	4·0
Perceived negative consequences	What are the disadvantages of using the HPS?	No disadvantage	26	52·0
		Cost of extra cleaning materials	11	22·0
		Takes up space inside the home	7	14·0
		Infant cries (from boredom)	7	14·0
		Extra item to clean	6	12·0
Perceived self-efficacy	What makes it easy for you to use the HPS?	Easy to assemble/rope easy to tie	27	54·0
		Weighs little/Easy to move (including mattress)	25	50·0
		Good size/Takes little space inside	22	44·0
		Door facilitates easy use	14	28·0
		Safe design/Infant easily visible	14	28·0
		Design encourages infant play (size/comfort)	13	26·0
		Bamboo structure strong/Stable/Durable	12	24·0
		Can be taken outside	8	16·0
		Older children who can watch infant	8	16·0

		Good width to slats to encourage standing	4	8·0
	What makes it difficult for you to use the HPS?	Difficult to rethread rope when dismantled	19	38·0
		No older children to watch infant	16	32·0
		Lack of toys	16	32·0
		Difficult to move outside/Heavy without help	8	16·0
		Rope may become loose/Structure falls	4	8·0
		Nothing	3	6·0
		Takes up space/House is small	2	4·0
		No older children to watch infant	2	4·0
		Plastic can get hot in sun	1	2·0
		Height insufficient	1	2·0
Access	What makes it easy for you to keep the HPS clean?	Plastic covering easily cleaned	39	78·0
		Mattress lightweight/Small to carry/Removable	38	76·0
		Requires little water	34	68·0
		Dries quickly (in/out of sun)	16	32·0
		Bamboo stays clean/easy to wipe	14	28·0
		Requires little soap	13	26·0
		Plastic does not absorb smell/urine/dirt	5	10·0
		Soap easy to buy/Inexpensive	2	4·0
		Water easily available	2	4·0
	What makes it difficult for you to keep the HPS clean?	Lack of/Expense of buying soap	28	56·0
		Water unavailable at times	13	26·0
		Requires extra cleaning materials/Associated cost	12	24·0
		Nothing	6	12·0
		Material (rough bamboo/rope/open seams)	5	10·0
Perceived social norms	Who are the people who approve of you using the HPS?	Neighbours	48	96·0
		Immediate family (parents, grandparents, siblings)	33	66·0
		HEW/HDA/Other government worker	24	48·0
		Husband	20	40·0
		Friends of parents	18	36·0
		Aunts/Uncles/Family-in-law	18	36·0
		Community members/Guests/Passers-by	11	22·0
		Labourer/Customers	3	6·0
	Who are the people who disapprove of you using the HPS?	Nobody	37	74·0
		Friends of parents	6	12·0
		Neighbours	4	8·0
		Community members/Colleagues/Customers	3	6·0
BA, Barrier Analysis; HPS, household playspace.				

S7. Number of samples positive for presumptive *Campylobacter* spp. under each category of colony count.

	Intervention (n=50)					Control (n=50)				
	0-100	101-250	251-999	>1000	>2000	0-100	101-250	251-999	>1000	>2000
Baseline	2	2	14	6	3	3	10	13	0	0
Two weeks	0	1	14	2	0	4	4	8	1	1
Four weeks	2	6	4	2	0	3	5	6	0	0

S8. Feeding of fresh or reheated foods prepared as recommended, across study groups and time points.

Baseline						
	Intervention (n=50)		Control (n=50)		Total (N=100)	
	n	%	n	%	n	%
Morning meal	30	60·0	43	86·0	73	73·0
Midday meal	34	68·0	38	76·0	72	72·0
Evening meal	33	66·0	42	84·0	75	75·0
All safely prepared	25	50·0	31	62·0	56	56·0
**Two weeks**						
	Intervention (n=50)		Control (n=50)		Total (N=100)	
	n	%	n	%	n	%
Morning meal	31	62·0	38	76·0	69	69·0
Midday meal	41	82·0	38	76·0	79	79·0
Evening meal	37	74·0	39	78·0	76	76·0
All safely prepared	19	38·0	28	56·0	47	47·0
**Four weeks**						
	Intervention (n=50)		Control (n=50)		Total (N=100)	
	n	%	n	%	n	%
Morning meal	37	74·0	36	72·0	73	73·0
Midday meal	30	60·0	30	60·0	60	60·0
Evening meal	31	62·0	37	74·0	68	68·0
All safely prepared	21	42·0	24	48·0	45	45·0

Feeding of fresh or reheated foods ’prepared as recommended’ if: Meals were prepared less than 2 hours before eating (not reheated); Meals were prepared less than 2 hours before eating which were reheated to ‘almost boiling’; Meals were prepared more than 2 hours before eating but reheated to ‘almost boiling’. ‘Not prepared as recommended’ if: Meals were prepared less than 2 hours before eating but only heated to ‘warm’; Meals were prepared more than 2 hours before eating and reheated to ‘warm’.

Figure
Click here to access/download;Figure;S Budge et al 2020_Figure 1_CONSORT_flow diagram.docx

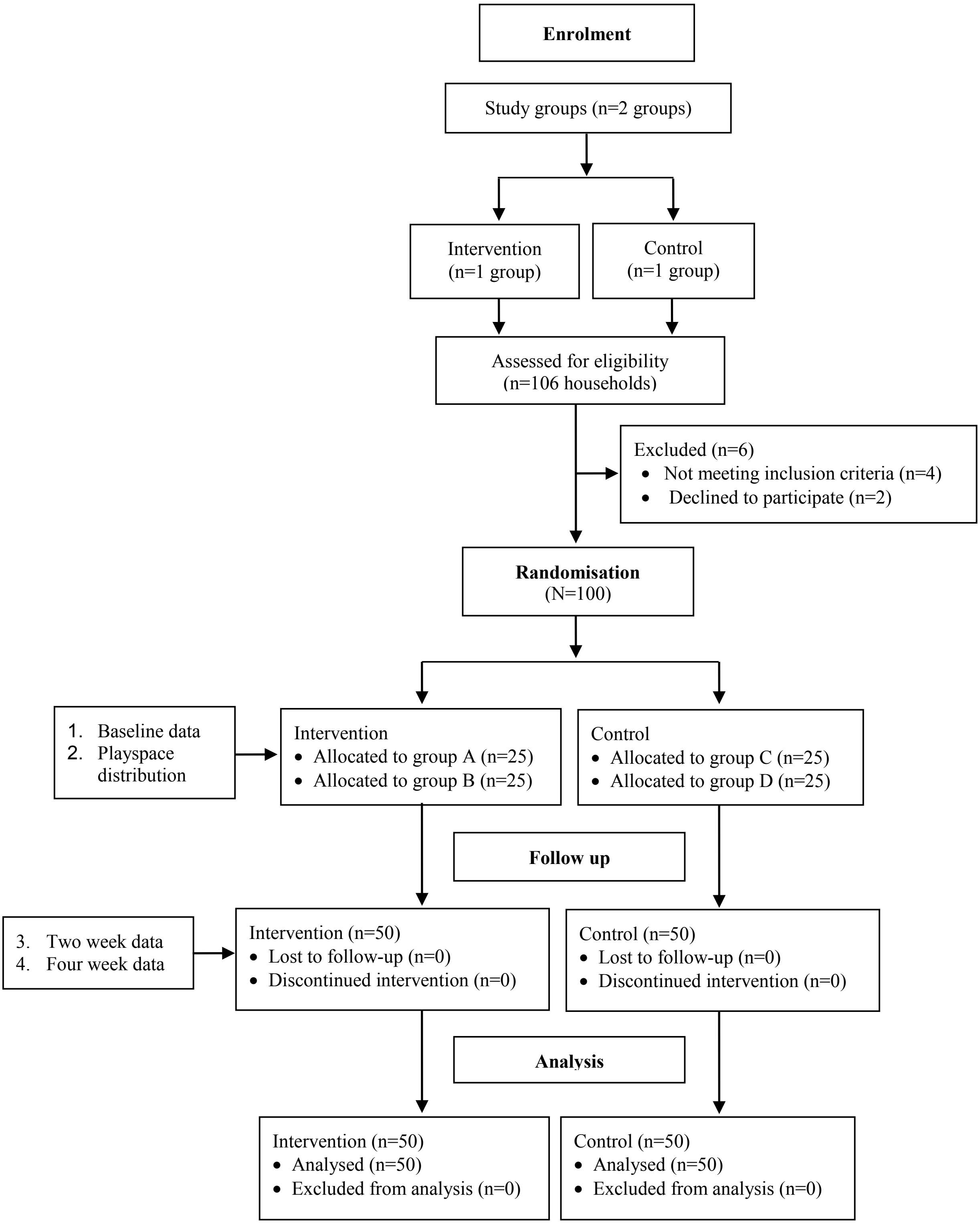

Figure 1. Modified version of CONSORT 2010 flow diagram of participants in the CAMPI feasibility trial.

Supporting Information

Click here to access/download
Supporting Information
S Budge et al 2020_CONSORT Checklist.doc
