# Supplementary material for: A randomised controlled feasibility trial of a BabyWASH household playspace: The CAMPI study
Source: PLoS Negl Trop Dis. 2021 Jul 14;15(7):e0009514. doi: 10.1371/journal.pntd.0009514 (PMC8312948; doi:10.1371/journal.pntd.0009514)
Supplement: Attachment — Submitted filename: PLOS NTDs Cover letter_Revision.docx [file pntd.0009514.s007.docx]

Dr Paul Hutchings

Faculty of Engineering and Physical Sciences

University of Leeds

Leeds

LS2 9JT, UK

[p.hutchings@leeds.ac.uk](mailto:p.hutchings@leeds.ac.uk)

26 January 2021

Dear Editor,

***A randomised controlled feasibility trial of a BabyWASH household playspace: The CAMPI study.***

I am pleased to submit a revised manuscript to PLOS NTDs. The reviewer comments and responses to them are provided in the following tables:

| **Manuscript: PNTD-D-20-01355** | | |
| --- | --- | --- |
| **Review comment** | **Response** | **Line number (revised doc)** |
| **General comments** | | |
| **Reviewer 1** | | |
| Interesting submission which is almost too voluminous in terms of information and results. 2 complementary papers highlighting the essential points would be interesting. but it is only a proposal | Thank you for the feedback. We appreciate that there is a lot of information in this paper. We had previous discussions in the team about writing two separate papers – one for feasibility outcomes and one for secondary health outcomes. However, as the study was a feasibility study it was not designed to be adequately powered to make strong claims on the health outcome data. We believe that included in this paper that health data can be considered as part of the broader assessment of feasibility, but we do not want to overclaim from that data by writing a separate paper on it. Hence, we included it in this study and believe that having both the primary and secondary outcomes together allows us to make the most comprehensive assessment of feasibility of a playspace as a hygiene intervention that has been published to date. | n/a |
| **Reviewer 2 (no response)** | | |
| **Reviewer 3** | | |
| Depending on the context, the above trial can be foundation for the need to address child welfare and growth monitoring in communities like in Ethiopia, however generalisation of the efficacy of the intervention other setting may significantly differ including also if the study lasted much longer in the same setting | Thank you for this comment. There is some mention of this within the limitations section with regards to the study being run by an NGO. However we have included another sentence to address this more directly. | 461-462 |
| **Methods** | | |
| **Reviewer 1** | No comments to address. Thank you for your feedback. | n/a |
| **Reviewer 2** | | |
| In the abstract it is stated: "households were identified and assessed for eligibility. Recruited households (N=100) were randomised (blinded) to intervention or control (both n=50)." Was the intervention blinded? This kind of intervention is difficult to blind. | Thank you for picking up on this detail. Participants were recruited blind prior to the trial start, but of course knew their status after collection of baseline data when playspaces were distributed. This is already addressed in line 111 and has now been clarified in the abstract. | 9 |
| I suggest to state what WASH data was collected. Was it infrastructure, access or use? Was it reported or observated? | This has been added into the methods section under ‘survey and anthropometry’. | 138-140 |
| Diarrhoea data collection might be limited by recall. Many studies observed that it could also be biased towards more cases for the ones without the intervention in order to claim for insfrastructure improvement. | Thank you for the valuable feedback. This has been added into the limitations section. | 416-417 |
| I suggest to specify faeces collection times in methods | This has been clarified under the heading ‘laboratory confirmation’. | 151-153 |
| **Reviewer 3** | | |
| The objectives have been stated as aims and the author can be advised to revisit | Thank you for clarifying this. The primary aim has been defined and the others as other objectives. Secondary outcomes remain as these. | 76-88 |
| The population had been described substantively | Thank you for your feedback. | n/a |
| **Results** | | |
| **Reviewer 1** | | |
| The sum of the available results makes it difficult to get to the point | Thank you for your comment. Table 2 provides an overall summary of the outcomes relating to the primary aim of the trial. With the adjusted edits from this revision we believe it is now clear what the primary results are and our interpretation of them. |  |
| Quality of figures and tables could be improved | We hope that during the proofing and publication process the clarity of figures and tables is suitable for viewing. | n/a |
| **Reviewer 2** | | |
| I suggest to describe water and sanitation outcomes since it is a relevant determinant of health and diarrhoea | Extra details from the WASH data in Table 1 have been added to this paragraph. | 229-230 |
| **Review comment** | **Response** | **Line number (revised doc)** |
| **Reviewer 3** | No comments to address. Thank you for your positive feedback. | n/a |
| **Conclusion** | | |
| **Reviewer 1** | No comments to address. Thank you for your feedback. | n/a |
| **Reviewer 2** | No comments to address. Thanks for your feedback. | n/a |
| **Reviewer 3** | | |
| Data supported the conclusion in some ways but the statement itself did not clearly elucidate this. Thus I have commented for the concluding statement to be revisited | Thank you. We agree this needs clarification. We hope that some added statements in the abstract, discussion and conclusion help to clarify this. | 19,20  362, 363  431  475 |
| Authors did made remarks on the positive observations from the intervention, however how data can be helpful is not explicitly discussed | We would like to politely rebuke that we believe the paper itself serves as a useful framework for similar trials. The discussion and limitations section provides suggested modifications for any future trials as well as necessary changes for a full-scale trial. | 478-479 |
| **Editorial and data presentation modification** | | |
| **Reviewer 1 (attachment)** | | |
| **Reviewer 2 (no comment)** | | |
| **Reviewer 3 (minor revision)** | | |
|  | | |
|  | | |
| **Reviewer comments (attached word document)** | | |
| **Review comment** | **Response** | **Line number (revised doc)** |
| **Major Comments** | | |
| **L26:** In relation to the reference provide for the statement that “Final height in adults results from many environmental factors which support growth in childhood”, I could not deduce the same meaning and I am curious to know where it had been stated, or do you imply Linear growth to be tandem to final height. | This sentence references a paper which examines this statement: ‘The stature of human adults reflects individual genotype and those environmental factors which influence child linear growth and limit the phenotypic expression of the genotype’. We have added to the sentence which hopefully helps clarify meaning. | 27 |
| **L40-42:** The message in this sentence needs to clarify what good health is defined as because the understanding may differ from the definition or the real meaning. | This has been clarified. | 41-42 |
| **L141-146:** Additional information is needed to elucidate all the essentials of the protocols or procedures involved. Documentation, packaging, transportation modalities from the field and average time from sample collection to testing etc should come out clearly. Were barcodes used, or forms filled for each specimen collected? | Thank you for the suggestion. As is referenced within the first line of the paragraph, sample collection in the trial followed a previously-validated methodology. Hence in the interest of space, we have not gone into full detail here. However we add some detail which we hope is sufficient. | 150-157 |
| **L453-460:** If a HPS remains a viable option to help reduce direct faecal-oral transmission and infant infection in these settings, what is the basis, given that not all *a priori* criteria were met? | Thank you for this comment. We believe that this requires a more nuanced interpretation of the data than as it stands. We believe the HPS remains a viable option given the encouraging acceptability and mixed adherence, but a full-scale trial is required to further assess efficacy on health outcomes; also, necessary adjustments would need to be made as are recommended. We have clarified this. | 19,20  362, 363  431  475 |
| **Minor Comments** | | |
| L8: What criteria were used for the identification and eligibility assessed for the 106 households: | Thank you, these criteria are discussed later in the methods section and we believe this level of detail is not necessary for the abstract. | 108-112 |
| L9-L10: Outcomes included recruitment, attrition, adherence, and acceptability (have been defined) | We do not think this comment is indicating a revision to be made. |  |
| L10-11: If data were collected at baseline, two and four weeks but why is data at baseline not presented? | Baseline data is presented in table 1. There was no playspace data available at baseline, as the playspace was distributed following baseline data collection. | Table 1 |
| L18: Despite failing some a priori criteria, the trial demonstrated good feasibility among intervention households: What do you consider as good feasibility and how was it defined. | This has been described under the paragraph ‘Implementation outcomes:  Evaluation of trial outcomes and proceeding with future definitive trial’ | 159-175 |
| L48: Give few examples of zoonotic disease which can be carried by domestic animals as reservoirs. | This has been added thank you for the suggestion. | 51-52 |
| L52-53: what is a household playspace in the context of this trial. This should be defined for the general reader comprehension of the term in context. | This has been clarified. Our previous paper which details the design and initial acceptability of a playspace is also referenced (23). | 56 |
| L54-55: Clarify how in rural areas, a HPS may offer some protection from infection during early growth periods. | This is explained in the previous sentence, but we have moved sentences around to provide better clarification. | 57-59 |
| L57-58: What were the key findings of the previous review of the evidence on the health and non-health benefits of a HPS or playmat | Thank you, we do not wish to go into too much detail here as the paper is referenced. Some detail has been added. | 61-63 |
| L78-79: Yes, the trial did not aim to determine the effect of the HPS on health outcomes as a possible hypothesis to measure but had the aim of establishing the feasibility of future definitive RCT to evaluate efficacy of a HPS. Therefore, a hypothesis can be provided and evaluated in relation to feasibility of future RCT and efficacy of a HPS. | Thank you, this has been clarified. | 76-88 |
| L97: How did eligible households became “eligible”, what was the eligibility criteria? | Thank you. The inclusion criteria are defined later in the paragraph. | 108-112 |
| L104: Is the inclusion criteria the same as the eligibility criteria? | Yes. This is clarified. | 108 |
| **Other Comments** | | |
| What is a **kebele** and it should be identified as a non-English word. | This is described. | 103 |
| The abbreviations; WASH and CAMPI, in long title should be written if full. | Thank you, we would like to refrain from writing in full in the title in the interest of space. Frequently, study names are not written in full in paper titles (e.g. <https://www.ncbi.nlm.nih.gov/pmc/articles/PMC4204608/>) | Title |
|  | | |
|  | | |
| **Reviewer response (attached PDF)** | | |
| **Review comment** | **Response** | **Line number (revised doc)** |
| Too many objectives, there must be a primary objective and secondary objectives. Perhaps divide your objectives into operational and health objectives. | Thank you, this has been edited into a primary aim and secondary outcomes accordingly. | 76-88 |
| Line 117 – add country | Region (SNNPR) and country (Ethiopia) added. | 121 |

In additional, following the request made during the technical check on resubmission we have added an additional Supplementary Table 9 that shows the data referenced on Line 432 (track changes version).

We thank you in advance for considering our manuscript.

Yours sincerely,

Dr Paul Hutchings
