# Supplementary material for: A randomised controlled feasibility trial of a BabyWASH household playspace: The CAMPI study
Source: PLoS Negl Trop Dis. 2021 Jul 14;15(7):e0009514. doi: 10.1371/journal.pntd.0009514 (PMC8312948; doi:10.1371/journal.pntd.0009514)
Supplement: Attachment — Submitted filename: PLOS NTDs Cover letter_Revision_3.docx [file pntd.0009514.s008.docx]

Dr Paul Hutchings

Faculty of Engineering and Physical Sciences

University of Leeds

Leeds

LS2 9JT, UK

[p.hutchings@leeds.ac.uk](mailto:p.hutchings@leeds.ac.uk)

26 May 2021

Dear Editor,

***A randomised controlled feasibility trial of a BabyWASH household playspace: The CAMPI study.* (PNTD-D-20-01355R1)**

I am pleased to submit a revised manuscript to PLOS NTDs. We believe these should satisfy the final comments provided by Reviewer 4 and hope that you can now accept this manuscript for publication.

| **Comment** | **Comment/change made** | **Page number** |
| --- | --- | --- |
| What criteria did authors use to determine 100 [households] is “sufficient”? | As this is a feasibility study the sample size was based on the data providing sufficient precision for the parameters of interest, rather than statistical power. A sample size of 100 was deemed sufficient since to achieves a maximum standard error of 0.05 for a proportion, which ensures a 95% confidence interval in the estimated results with a maximum width of +/-10%. This was deemed achievable with the resources available and is in line with recommendations for feasibility studies where the parameter of interest is a proportion. | 98-104 |
| Can the authors please clarify here which confidence intervals (e.g., approximate, exact) were calculated and reported? | Estimated confidence intervals were calculated for descriptive statistics. The Wald confidence interval is estimated. | 181, 182 |
| Was the study powered to conduct this analysis [GEE model] and achieve significant differences or is this being done for preliminary/exploratory purposes only? The authors do comment on the limitations in the discussion so it may be helpful to emphasize this when presenting the results. | As this is a feasibility study, in line with the CONSORT extension for feasibility studies, we do not present p-values. We discuss the limitations of the small sample size throughout the manuscript. We try to emphasise that the results were exploratory as this is a feasibility trial. This has been added to the results. | 196-197  233-234  410-411 |
| In the statistical analysis section, the authors write, “Results are expressed as populated averaged odds ratios (ORs) with 95% CI.” Can they please clarify which confidence intervals were reported here? The same comment for the statement, “Change in diarrhoeal and Campylobacter prevalence between study groups was estimated using a GEE intercept-only model with OR and 95% CI.” Table 4 is the only place where Wald is specified, so clarification would be helpful given the small sample size. | Again this is the Wald confidence interval, this has been addressed above. | 182, 208 |

In addition, we made the final adjustments required by the editorial office in terms of a Legend for the supplementary information and changes within the submission form.

We thank you in advance for considering our manuscript.

Yours sincerely,

Dr Paul Hutchings
