# Supplementary material for: A randomised controlled feasibility trial of a BabyWASH household playspace: The CAMPI study
Source: PLoS Negl Trop Dis. 2021 Jul 14;15(7):e0009514. doi: 10.1371/journal.pntd.0009514 (PMC8312948; doi:10.1371/journal.pntd.0009514)
Supplement: Attachment — Submitted filename: Reviewer Comments.docx [file pntd.0009514.s006.docx]

**Long title: A randomised controlled feasibility trial of a Baby WASH household playspace: The CAMPI study**

**Short title:** A randomised controlled feasibility trial of a household playspace

**Major Comments:**

**L26: ^(1)^** In relation to the reference provide for the statement that “Final height in adults results from many environmental factors which support growth in childhood”, I could not deduce the same meaning and I am curious to know where it had been stated, or do you imply Linear growth to be tandem to final height.

**L40-42:** The message in this sentence needs to clarify what good health is defined as because the understanding may differ from the definition or the real meaning.

**L141-146:** Additional information is needed to elucidate all the essentials of the protocols or procedures involved. Documentation, packaging, transportation modalities from the field and average time from sample collection to testing etc should come out clearly. Were barcodes used, or forms filled for each specimen collected?

**L453-460:** If a HPS remains a viable option to help reduce direct faecal-oral transmission and infant infection in these settings, what is the basis, given that not all *a priori* criteria were met?

**Minor Comments**

L8: What criteria were used for the identification and eligibility assessed for the 106 households:

L9-L10: Outcomes included recruitment, attrition, adherence, and acceptability (have been defined)

L10-11: If data were collected at baseline, two and four weeks but why is data at baseline not presented?

L18: Despite failing some a priori criteria, the trial demonstrated good feasibility among intervention households: What do you consider as good feasibility and how was it defined.

L48: Give few examples of zoonotic disease which can be carried by domestic animals as reservoirs.

L52-53: what is a household playspace in the context of this trial. This should be defined for the general reader comprehension of the term in context.

L54-55: Clarify how in rural areas, a HPS may offer some protection from infection during early growth periods.

L57-58: What were the key findings of the previous review of the evidence on the health and non-health benefits of a HPS or playmat

L78-79: Yes, the trial did not aim to determine the effect of the HPS on health outcomes as a possible hypothesis to measure but had the aim of establishing the feasibility of future definitive RCT to evaluate efficacy of a HPS. Therefore, a hypothesis can be provided and evaluated in relation to feasibility of future RCT and efficacy of a HPS.

L97: How did eligible households became “eligible”, what was the eligibility criteria?

L104: Is the inclusion criteria the same as the eligibility criteria?

**Other Comments**

1. What is a **kebele** and it should be identified as a non-English word.
2. The abbreviations; WASH and CAMPI, in long title should be written if full.
